# Rolosense: Mechanical Detection of SARS-CoV-2 Using a DNA-Based Motor

**DOI:** 10.1021/acscentsci.4c00312

**Published:** 2024-05-21

**Authors:** Selma Piranej, Luona Zhang, Alisina Bazrafshan, Mariana Marin, Gregory B. Melikian, Khalid Salaita

**Affiliations:** † Department of Chemistry, Emory University, Atlanta, Georgia 30322, United States; ‡ Department of Pediatrics, Emory University School of Medicine, Atlanta, Georgia 30322, United States; § Children’s Healthcare of Atlanta, Atlanta, Georgia 30322, United States; ∥ Wallace H. Coulter Department of Biomedical Engineering, Georgia Institute of Technology and Emory University, Atlanta, Georgia 30322, United States

## Abstract

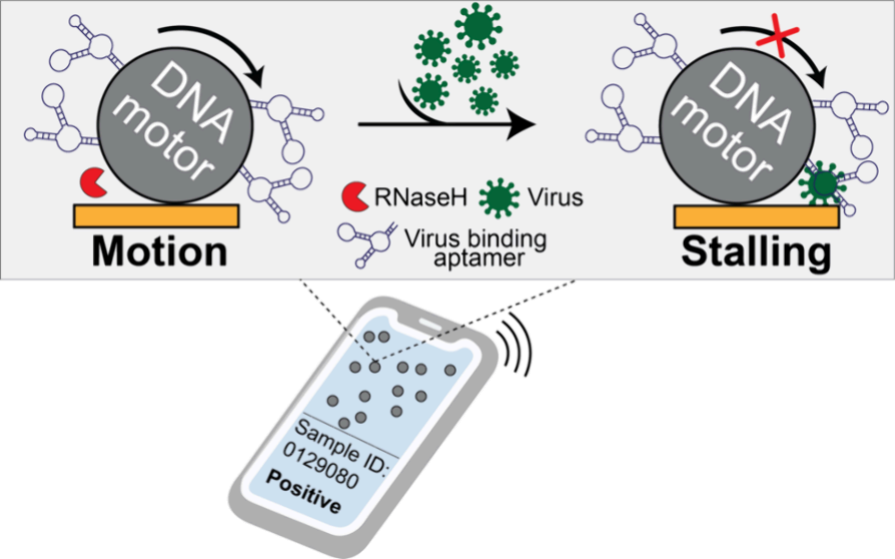

Assays that detect viral infections play a significant role in limiting the spread of diseases such as SARS-CoV-2. Here, we present Rolosense, a virus sensing platform that leverages the motion of 5 μm DNA-based motors on RNA fuel chips to transduce the presence of viruses. Motors and chips are modified with aptamers, which are designed for multivalent binding to viral targets and lead to stalling of motion. Therefore, the motors perform a “mechanical test” of the viral target and stall in the presence of whole virions, which represents a unique mechanism of transduction distinct from conventional assays. Rolosense can detect SARS-CoV-2 spiked in artificial saliva and exhaled breath condensate with a sensitivity of 10^3^ copies/mL and discriminates among other respiratory viruses. The assay is modular and amenable to multiplexing, as demonstrated by our one-pot detection of influenza A and SARS-CoV-2. As a proof of concept, we show that readout can be achieved using a smartphone camera with a microscopic attachment in as little as 15 min without amplification reactions. Taken together, these results show that mechanical detection using Rolosense can be broadly applied to any viral target and has the potential to enable rapid, low-cost point-of-care screening of circulating viruses.

## Abstract 2

Rolosense is a rapid, label-free diagnostic tool employing DNA-based motors with multivalent binding to mechanically identify respiratory viruses with the simplicity of smartphone camera readouts.

Virus sensing is primarily performed using nucleic acid-based assays or alternatively by detecting protein antigens using colorimetric, fluorogenic, or electrochemical reporters. The gold-standard diagnostic for SARS-CoV-2 infection is RT-qPCR, which detects down to ∼10^2^–10^3^ viral RNA copies/mL and is typically performed at central facilities with a 10–15 h turnaround time.^1,2^ Alternate nucleic acid-based diagnostics include loop-mediated isothermal amplification (LAMP),^3−5^ recombinase polymerase amplification (RPA),^6−8^ and the integration of CRISPR-Cas systems,^9−12^ which do not require special instrumentation but can suffer from nonspecific amplification under isothermal conditions leading to false-positive results. On the other hand, protein antigen tests which work by capturing viral proteins on immobilized antibodies, such as the lateral flow assay (LFA), are less sensitive (10^4^–10^6^ copies/mL) but fairly simple to use and result in short turnaround times.^13−16^ Nonetheless, this has led to their wide adoption, as LFA tests can be performed at home without the need for bulky temperature-control or spectrophotometer instruments. Developing new platforms that combine the sensitivity of RT-qPCR with the simplicity and fast turnaround time of LFAs is needed to address the current and future pandemics.

One unexplored approach for chemical sensing pertains to the mechanical testing of an analyte. For example, single-molecule force spectroscopy methods, such as optical tweezers,^17,18^ atomic force microscopy (AFM),^19,20^ and tip-enhanced Raman spectroscopy,^21^ can identify single virus–ligand interactions with high fidelity. Thus, mechanical testing of a virus offers an alternate strategy for detection with exquisite sensitivity. Unfortunately, using force spectroscopy for analytical sensing is prohibitive because of the serial nature of these methods, interrogating one molecule at a time, and the need for expensive and dedicated instrumentation. Autonomous force-generating motors that can be characterized in parallel may offer an alternate approach to using mechanotransduction for analytical sensing.

Herein we present a mechanical-based viral sensing platform termed Rolosense to detect whole intact SARS-CoV-2 particles. Rolosense is a label-free and amplification-free approach, which is advantageous because such methods reduce cost and, in our case, simplify instrumentation needed for readout avoiding fluorescence dyes and spectrometers that are commonly used for nucleic acid-based assays. Our assay uses DNA-based motors that function as the mechanical transducer reporting on specific target binding events (Figure 1a). We leveraged our recent work using these motors to detect and transduce DNA logic operations.^22^ The motor consists of a DNA-coated spherical particle (5 μm diameter) that hybridizes to a surface modified with complementary RNA. The particle moves with speeds of >1 μm/min upon addition of ribonuclease H (RNaseH), which selectively hydrolyzes duplexed RNA and ignores single-stranded (ss) RNA.^23^ The DNA motor which can be comprised of microparticles,^57^ nanoparticles,^58^ and DNA origami^56^ consumes chemical energy stored in the RNA chip to generate piconewton mechanical work.^28,45^ To detect the SARS-CoV-2 virus, the DNA motors and the RNA chip are modified with virus-binding ligands (i.e., aptamers) with high affinity for the S1 subunit of spike protein that is abundantly displayed on each virion.^24^ Virus binding to both the motor and the chip surface leads to motor stalling (Figure 1a). In other words, the microparticle moves along the surface through a “cog-and-wheel” mechanism, and only the SARS-CoV-2 viral target acts as a “wrench” to inhibit this activity. The work discussed here, using motion-based sensing via DNA-based motors, introduces a different approach to chemical sensing to test whether far-from-equilibrium sensing provides enhanced sensitivity over conventional binding assays. Conventional analytical techniques involve chemical measurements at or near equilibrium, while chemical sensing in biology typically occurs at far-from-equilibrium conditions. Far-from-equilibrium sensing in biology outperforms conventional methods by not relying on equilibria. It takes advantage of nonlinear processes, akin to the amplification observed in biological examples such as bacterial chemotaxis,^25^ where bacteria continuously sense and respond to nutrient gradients, kinase pathways that can rapidly amplify growth factor signals,^26^ and the T cell receptor (TCR) mechanisms for antigen recognition, which involve highly sensitive and specific activation without the equilibrium constraints.^27^ In addition, unlike current nucleic acid and protein assays we do not need fluorescence or absorbance measurements to detect a target of interest. Instead, detection of the viral target occurs when the *mechanical force* generated by the motor (∼100 pN) is insufficient to overcome the mechanical stability of the aptamer–target complex.^28^ This binding event is transduced in a label-free fashion by measuring the displacement of the motor. Another key advantage of Rolosense is its ability to multiplex and detect multiple respiratory viruses in the same assay. This will be critical in patient care and in minimizing false-positive results due to similar symptoms.

**Figure 1:**
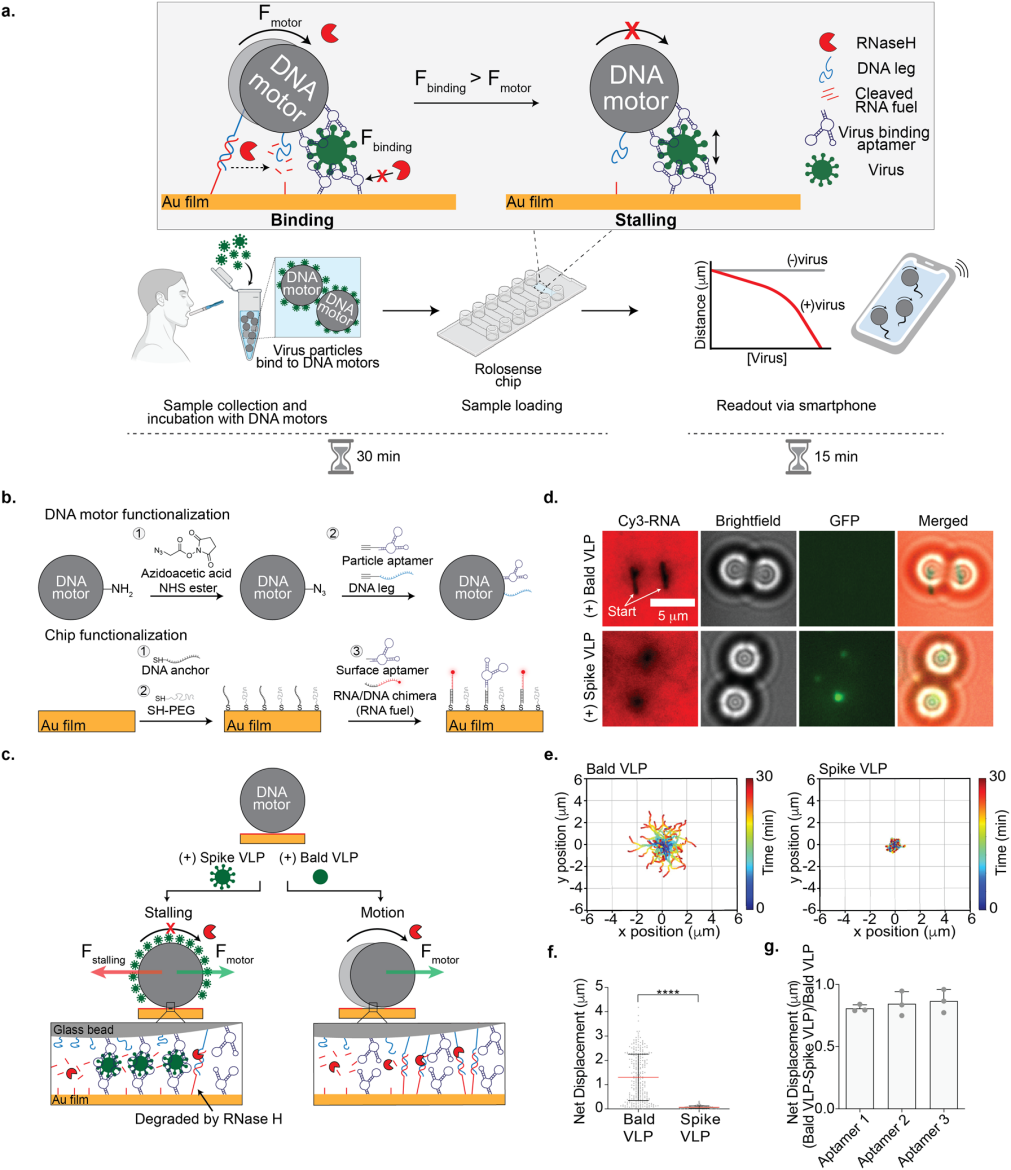
**Optimizing Rolosense with GFP-labeled virus-like particles (VLPs).** (a) Schematic workflow of the Rolosense assay. The motors multivalently bind to virus particles. The presence of virus particles leads to motor stalling, which reduces the net displacement or distance traveled by the motors. Readout can be performed using simple bright-field time-lapse imaging of the motors. In principle, readout can be performed in as little as 15 min using a smartphone camera. (b) Schematic of DNA motor and chip functionalization. The DNA motors were modified with a binary mixture of DNA leg and aptamers that have high affinity for the SARS-CoV-2 spike protein. The Rolosense chip is a gold film also composed of two nucleic acids: the RNA/DNA chimera, which is referred to as the RNA fuel, and the same aptamer as the motor. (c) Schematic of the detection of the SARS-CoV-2 virus. In the presence of VLPs expressed with spike protein (spike VLPs), the motors stall on the Rolosense chip following the addition of the RNaseH enzyme, as the stalling force (red arrow) is greater than the force generated by the motor (green arrow). When incubated with the bald VLPs or VLPs lacking the spike protein, the motors respond with motion and roll on the chip in the presence of RNaseH. (d) Bright-field and fluorescence imaging of DNA motors detecting GFP-labeled spike VLPs. The RNA fuel was tagged with Cy3, shown here in red. Motors were incubated with 25 pM GFP-labeled bald and spike VLPs diluted in 1× PBS. Samples with GFP-labeled spike VLPs show stalled motors and no Cy3 depletion tracks, in contrast to samples of GFP-labeled bald VLPs. Note that stalled motors often showed GFP signal colocalization. (e) Plots showing the trajectory of motors with bald and spike VLPs. All of the trajectories are aligned to 0,0 (center) of the plots for time = 0 min. Color indicates time (0 → 30 min). (f) Plot showing net displacement of over 100 motors incubated with 25 pM bald and spike VLPs. The error bars and the red lines represent the standard deviation and the mean of the distribution, respectively. **** indicates *P* < 0.0001. Experiments were performed in triplicate. (g) Plot showing the difference in net displacement between the bald and spike VLPs normalized by the bald VLP displacement in conditions using different aptamers. Each data point indicates the pooled average for an independent experiment. Error bars show the standard deviation.

Using motors modified with multivalent binding of aptamers that have a high affinity for spike protein, we were able to demonstrate a limit of detection (LoD) of 10^3^ viral copies/mL for SARS-CoV-2 B.1.617.2 and BA.1 in artificial saliva and in exhaled breath condensate without any sample preparation or amplification steps. Note that these were the variants available to us over the course of this study. We demonstrate specific detection of SARS-CoV-2 viral particles, as our motors do not respond to influenza A or other human corona viruses such as OC43 and 229E. We also show the ability to multiplex by detecting influenza A and SARS-CoV-2 in the same “pot”. Our assay can be read out via smartphone microscope in as little as 15 min. Overall, Rolosense enables rapid, sensitive, and multiplexed viral detection for disease monitoring.

## Design Principles of the Rolosense Platform

We functionalized DNA-based motors and chips with DNA aptamers reported in the literature that had high affinity for spike protein (S1) (Supplementary Table 1 and Figure S1).^29,30^ Aptamers as virus-binding ligands have several advantages, such as ease of storage, long-term stability, and lower molecular weight. We started our investigations with a 50 nt S1 aptamer, aptamer 1. As depicted in Figure 1b, the amine-modified motors were functionalized and coated with a binary mixture of both the DNA leg and aptamer 1. The planar Rolosense chip was modified with a binary mixture of Cy3-labeled RNA fuel and aptamer 1 (Figure S2). The oligonucleotides were tethered to the surface by hybridization to a monolayer of 15-mer ssDNA, which we call the DNA anchor. Tuning the ratio of the DNA legs/RNA fuel to the aptamer was critical, as there is a trade-off between multivalent avidity to the virion and efficient motor motion.^22^ For example, high densities of aptamers lead to efficient virus binding but hamper processive motion. Conversely, low aptamer densities diminish virus binding but enhance motor speed and processivity. Accordingly, we screened different ratios of the aptamer/DNA leg on the particle and the aptamer/RNA fuel on the chip and measured the motor net displacement over a 30 min time window. We found that the introduction of an aptamer at 10% density or greater on the particle or the planar surface led to a significant reduction in motor displacements (Figure S3). Also, the motor displacement was more sensitive to the aptamer density on the spherical particle compared to that on the planar surface. Our results suggested that the optimal aptamer densities were 10% for the particle and 50% for the planar surface, as these motors showed 1.95 ± 0.97 μm net displacement over a 30 min time window compared to the no aptamer control, in which the motors traveled 2.56 ± 1.17 μm in 30 min (Figure S3). Based on these results, all subsequent experiments were conducted using motors and chips modified with 10% and 50% aptamer density, respectively.

We first tested our assay using GFP-tagged virus-like particles (VLPs) expressing the trimeric spike protein. We used noninfectious SARS-CoV-2 S D614G HIV-1 VLPs (spike VLPs) (Figure S4). As a control to test for cross-reactivity, we used GFP-tagged HIV-1 particles that lacked spike proteins (bald VLPs). The motor surface was functionalized with 10% aptamer 1 and the chip surface with 50% aptamer 1.^29^ The VLPs were incubated with the aptamer-functionalized DNA-based motors in 1× phosphate-buffered saline (PBS) for 30 min at room temperature. After 30 min, the DNA-based motors were washed via centrifugation (15,000 rpm, 1 min) and then added to the Rolosense chip that was also coated with the same aptamer. In the presence of RNaseH, the DNA-based motors incubated with the spike VLPs remained stalled on the surface (Figure 1c). The VLPs were likely sandwiched between the DNA-based motor and the chip surface, and this binding led to a stalling force that halted the motion. In contrast, DNA-based motors incubated with the bald VLPs lacking the spike protein translocated on the surface, which is expected because the bald VLPs do not bind to the aptamers. This was confirmed by optical and fluorescence microscopy (Figure 1d). Motors incubated with the bald VLPs displayed micrometer-length depletion tracks in the Cy3-RNA monolayer. The lack of fluorescence signal in the GFP channel indicates that there was minimal or no binding of bald VLPs to the motors. On the contrary, motors incubated with spike VLPs did not display Cy3-RNA depletion tracks, and the GFP fluorescence channel showed puncta colocalized with the stalled motors, confirming that the stalling was due to binding of spike VLPs. Bright-field real-time particle tracking also validated this conclusion. We observed long trajectories and net displacements greater than 1.5 μm for motors incubated with bald VLPs. The spike-VLP-incubated motors, on the other hand, displayed short trajectories with abrupt red puncta indicating stalling events and sub-1 μm net displacements (Figure 1e,f). Control motors without VLPs showed greater displacements than that of the bald VLP samples (Figure S5), likely due to nonspecific bald VLP binding. Regardless, these results demonstrate that the Rolosense design and mechanism for viral detection are valid and further motivated our subsequent experiments.

In principle, Rolosense is not unique to aptamers, and virtually any virus-binding ligand could be used for viral sensing. That said, in preliminary screens with two commercial antibodies, we found motor stalling with bald VLPs, suggesting issues with specificity (Figure S6). We thus focused efforts on screening across different aptamers reported to display high affinity and specificity for SARS-CoV-2 S1. Specifically, aptamers 1, 2, and 3^29,30^ have reported *K*_D_ values in the low-nanomolar range for S1: 39 nM for aptamer 1 and 13 nM for aptamer 2 (the *K*_D_ for aptamer 3 has not been reported, but we assume a similar low-nanomolar affinity, as it is a truncated version of aptamer 2 with similar activity). Using motors and surfaces functionalized with each of these aptamers (10% motor and 50% chip), we showed that aptamer 3 was the most sensitive and specific for Rolosense (Figure 1g). Based on these data, we performed all subsequent experiments using aptamer 3 unless noted otherwise.

## Detecting SARS-CoV-2 in Artificial Saliva

**Figure 2:**
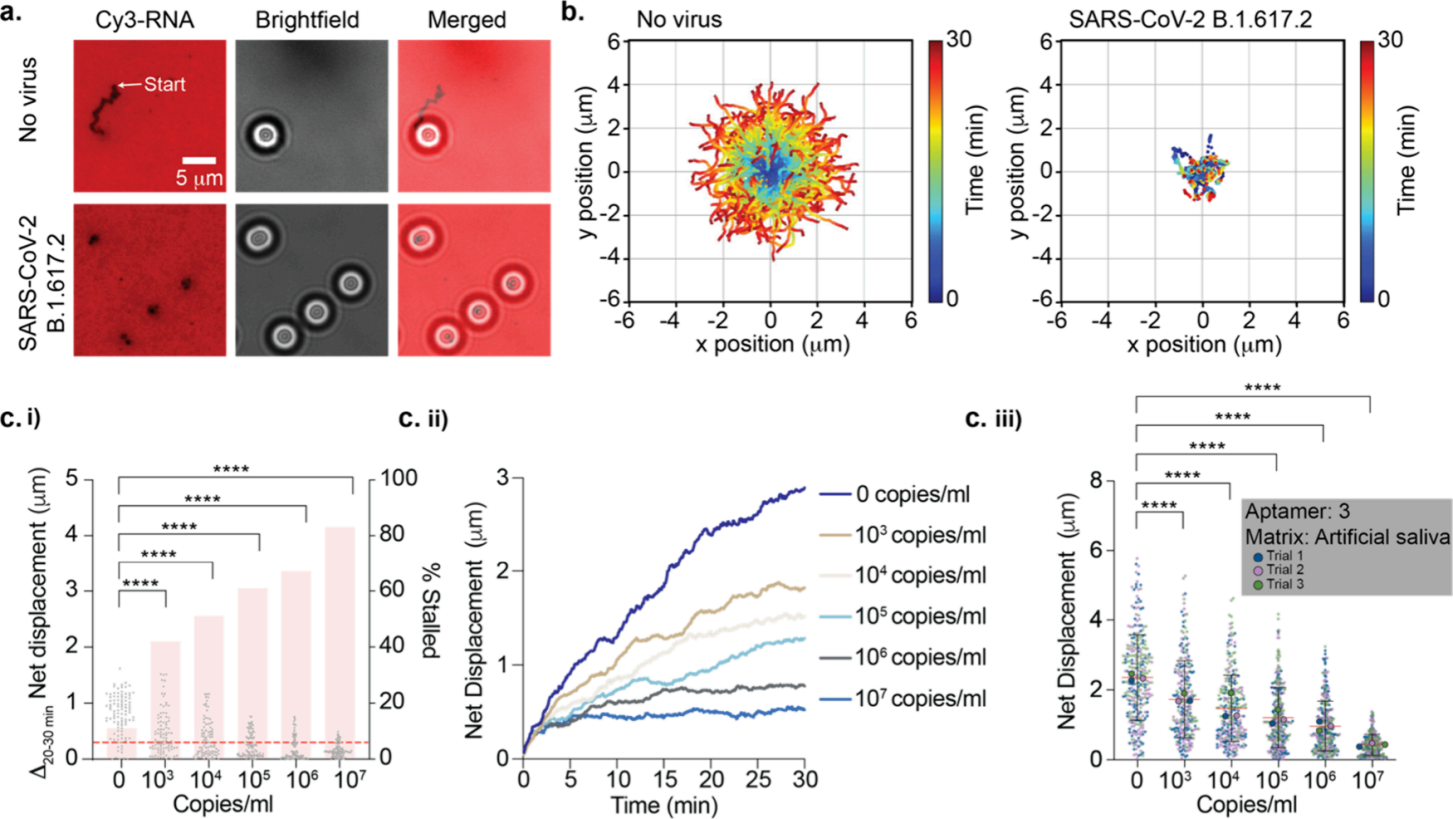
**Detecting SARS-CoV-2 virus in artificial saliva.** (a) Fluorescence and bright-field imaging of DNA motors detecting the presence of 10^7^ copies/mL UV-inactivated SARS-CoV-2 B.1.617.2 spiked in artificial saliva. DNA motors were incubated for 30 min with the virus samples. Samples with SARS-CoV-2 show stalled motors and no depletion tracks, in contrast to samples lacking the virus. (b) Plots showing the trajectories of motors with no virus and 10^7^ copies/mL UV-inactivated SARS-CoV-2 B.1.617.2 strain spiked in artificial saliva. All the trajectories are aligned to 0,0 (center) of the plots for time = 0 min. Color indicates time (0 → 30 min). (c) (i) Plots of the Δnet displacement as well as the percentage of motors stalled in the final 10 min of the 30 min time-lapse (*t* = 20–30 min) for 100 motors incubated with various concentrations of SARS-CoV-2 B.1.617.2. To calculate a percentage of stalled motors, a 0.300 μm threshold was used (red dashed line). (ii) Plots of the net displacement over the 30 min time-lapse for 30 motors incubated with different concentrations of SARS-CoV-2 B.1.617.2. (iii) Superplots of net displacement of over 300 motors for three independent replicates. Each motor is color-coded based on the triplicate data: blue for trial 1, pink for trial 2, and green for trial 3. The mean for each trial is superimposed on top of the plots. The error bars and the red lines represent the standard deviation and the mean of the distribution, respectively. UV-inactivated SARS-CoV-2 B.1.617.2 samples were spiked in artificial saliva and incubated with motors functionalized with aptamer 3 at room temperature for 30 min. **** indicates *P* < 0.0001.

We then aimed to validate our Rolosense assay using an authentic SARS-CoV-2 virus that was UV-inactivated. We tested the SARS-CoV-2 B.1.617.2 strain, known as the Delta variant. For these sets of experiments, we spiked the virus into artificial saliva and performed Rolosense for a viral readout. First, we wanted to test whether the motors and the Rolosense assay could tolerate the artificial saliva matrix, since it contains mucins and divalent ions such as calcium that may interfere with the assay. Motors were suspended in artificial saliva for 30 min and then added to the aptamer-decorated chip for a readout. We found that motion was not affected by the artificial saliva matrix, as the motors displayed long trajectories with the addition of RNaseH enzyme and the average net displacement (2.20 ± 1.38 μm) was comparable to that of controls performed in 1× PBS (2.97 ± 1.40 μm) (Figure S7). Once we validated the assay in artificial saliva, we next incubated motors functionalized with 10% aptamer 3 with 10^7^ copies/mL SARS-CoV-2 B.1.617.2 for 30 min at room temperature. After 30 min, the DNA-based motors were washed via centrifugation (15,000 rpm, 1 min) and then added to the Rolosense chip presenting aptamer 3. In the presence of RNaseH, the motors remained stalled on the surface and did not display depletion tracks (Figure 2a). Control motors without virus displayed long depletion tracks in the Cy3-RNA channel. Bright-field particle tracking confirmed these results, as we observed hampered particle trajectories for the SARS-CoV-2 B.1.617.2 condition compared to the long trajectories displayed by motors without any virus (Figure 2b). Similar results were observed with the original SARS-CoV-2 strain first isolated in the U.S., in the State of Washington and hence described here as the Washington (WA-1) strain (Figure S8a,b). In a control experiment in which we withheld the surface aptamer, we observed that aptamer-presenting motors incubated with 10^7^ copies/mL SARS-CoV-2 WA-1 displayed long net displacements and depletion tracks in the Cy3-RNA channel (Figure S9). This confirmed that the stalling observed in the presence of a virus is due to virus particles bridging the aptamers on the bead to the aptamers on the chip. To optimize the workflow of the Rolosense assay, we tested whether we could forego the washing step following motor incubation with virus. Our results indicated that running the assay without the wash step does not degrade the integrity of the chip, as the RNA on the surface remained intact (Figure S10). We also observed similar net displacements between the motors with and without wash when incubated with 10^7^ copies/mL WA-1 virus. In addition, we tested whether decreasing the virus sample incubation time affected the performance of Rolosense. We found that decreasing the incubation time with the motors down to 10 min did not impact the performance of the Rolosense assay, as the majority of the motors remained stalled (Figure S11).

Next, we aimed to determine the limit of detection (LoD) of the Rolosense assay in artificial saliva. Because the ensemble average net displacement of many motors conceals the time-dependent process of virus encounter, especially as we approach the LoD, we analyzed the fraction of stalled motors as well as the change in net displacement in the final 10 min of our time-lapse and compared that to the initial 20 min. From bright-field particle tracking, we observed that increasing the SARS-CoV-2 B.1.617.2 concentration results in an increase in the percentage of stalled motors (*N* = 100) (Figure 2c(i)). The threshold used to identify stalled motors was selected at 0.300 μm, as that is the net displacement for immobile particles and is likely the result of drift and localization error. The difference in motor net displacement over the *t* = 20–30 min window compared to that of the *t* = 0–20 min window showed a significant decrease with increasing virus concentration down to 10^3^ copies/mL (*P* < 0.0001). Further supporting these findings, we observed a decrease in net displacement over time with increasing virus concentration for *N* = 30 motors (Figure 2c(ii)). As motors incubated with virus probe the chip surface, they will eventually stall when a virus bridges the motor and chip, resulting in a decrease in motion over time (Figure S12). Therefore, the detection mechanism is not deterministic but based on chance encounters between the motors with virus and the aptamers on the chip. This means that the likelihood of motor stalling does not increase linearly with the concentration of the virus; instead, it is subject to randomness and the rate of encounters. To best understand how motors behave when they encounter a virus, we performed modeling experiments in which we varied the virus concentration and observed a range of outcomes based on different step sizes and paths, reflecting the randomness of real-life molecular interactions (Figure S13). Indeed, our modeling confirms a distribution in the motion of motors, where some motors never encounter the virus and continue moving while others do encounter the virus and consequently stall. This approach emphasizes that the encounter rate of the motors with the virus and aptamer on the chip is a matter of chance and is not linearly dependent on the virus concentration, leading to a gradual and varied motor response.

Taken together, in triplicate experiments we demonstrated an LoD of 10^3^ copies/mL for SARS-CoV-2 B.1.617.2 (Figures 2c(iii) and S14). We also determined the LoD for the Rolosense assay with other SARS-CoV-2 variants such as Washington (WA-1) and Omicron (BA.1) spiked in artificial saliva. The Rolosense assay showed a sensitive response to WA-1 but not BA.1 with an LoD of ∼10^3^–10^4^ copies/mL (Figures S8c, S14, and S15). The LoD for B.1.617.2 and WA-1 was greater than that for the BA.1 variant, which was expected given that aptamer 3 was selected using S1 of the initial Wuhan strain. Interestingly, the mutations in S1 for the B.1.617.2 strain primarily led to an increase in the net positive charge of the protein,^31^ which likely aids in enhancing binding to a negatively charged aptamer. The LoD for the BA.1 strain is weaker, but this is expected given the increased number of mutations in this most recent variant. Importantly, Rolosense demonstrates an LoD that is better than that of typical LFAs but using a DNA motor.^32,33^

**Figure 3:**
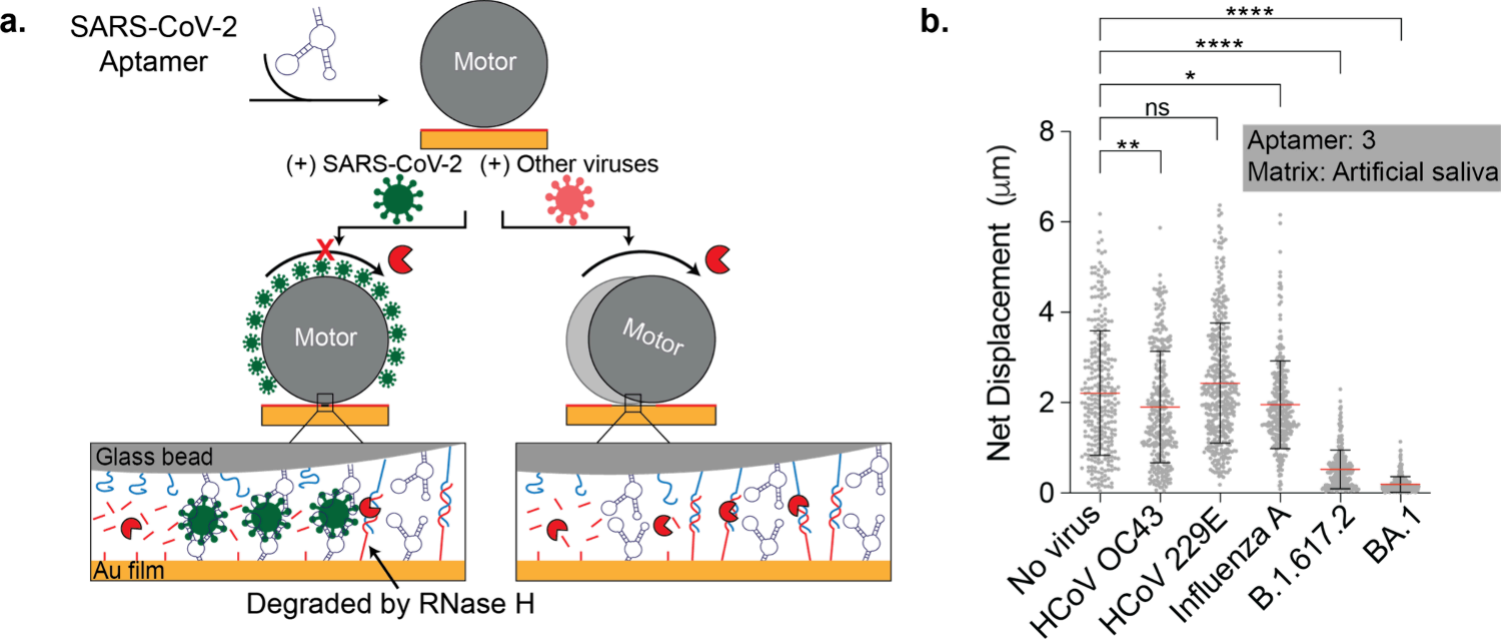
**Motors demonstrate a specific response to SARS-CoV-2 viruses.** (a) Schematic of motors modified with SARS-CoV-2 aptamer stalling when incubated with SARS-CoV-2 virus particles, which is in contrast to motors incubated with other viruses. (b) Plot showing the net displacement for over 100 motors incubated with 10^7^ copies/mL UV-inactivated HCoV OC43, HCoV 229E, influenza A, SARS-CoV-2 B.1.617.2, and SARS-CoV-2 BA.1 spiked in artificial saliva. The motors were functionalized with aptamer 3 and incubated for 30 min with each sample. All measurements were performed in triplicate. The error bars and the red lines represent the standard deviation and the mean of the distribution, respectively. ns, *, **, and **** indicate not statistically significant, *P* < 0.05, *P* < 0.01, and *P* < 0.0001, respectively.

To test for cross-reactivity and specificity of our assay, we measured the response of the motors incubated with other respiratory viruses, such as the seasonal common cold viruses HCoV OC43 and 229E, as well as the influenza A virus. These respiratory viruses present similar symptoms as the SARS-CoV-2 virus, and thus, it is important to distinguish between them. These samples were prepared in a manner similar to that of the SARS-CoV-2 variants and spiked in artificial saliva to run the Rolosense assay. As depicted in Figure 3a, the motors displayed high specificity and responded with motion to HCoV OC43, HCoV 229E, and influenza A, which is in direct contrast to the stalling observed in the presence of SARS-CoV-2 viruses (Figures 3b and S16). These data, along with the LoD data, confirm that the Rolosense assay exhibits a sensitive and specific response to the SARS-CoV-2 virus which is ultimately the result of the sensitivity and specificity of aptamer 3 to its SARS-CoV-2 target.

## Multiplexed Detection of SARS-CoV-2 and Influenza A Viruses

Given the need for distinguishing between a variety of respiratory viruses, we next aimed to test whether Rolosense can detect other viruses, such as the influenza A virus. This is well-suited for Rolosense, as the assay is modular and can easily be programmed to detect other whole virions. We created an influenza A motor by modifying it with 10% influenza A aptamer reported in the literature (*K*_D_ = 55 nM), with the chip presenting 50% aptamer.^34^ Following the protocol for SARS-CoV-2, the motors were incubated with different concentrations of the influenza A virus spiked in 1× PBS for 30 min. Although the motors stalled in the presence of high concentrations of influenza A virus such as 10^10^ copies/mL, the assay performed poorly in detecting low copy numbers (Figure S17). To address this issue, we supplemented the 1× PBS solution with 1.5 mM Mg^2+^ since divalent cations aid in secondary structure formation of aptamers.^35^ As expected, the assay improved with the addition of Mg^2+^, and we were able to detect as low as 10^4^ copies/mL influenza A virus using this aptamer. This suggests potential for Rolosense to detect influenza A infections, as a typical swab of patients with influenza yields ∼10^8^ genome copies/mL as estimated by PCR.^36^

**Figure 4:**
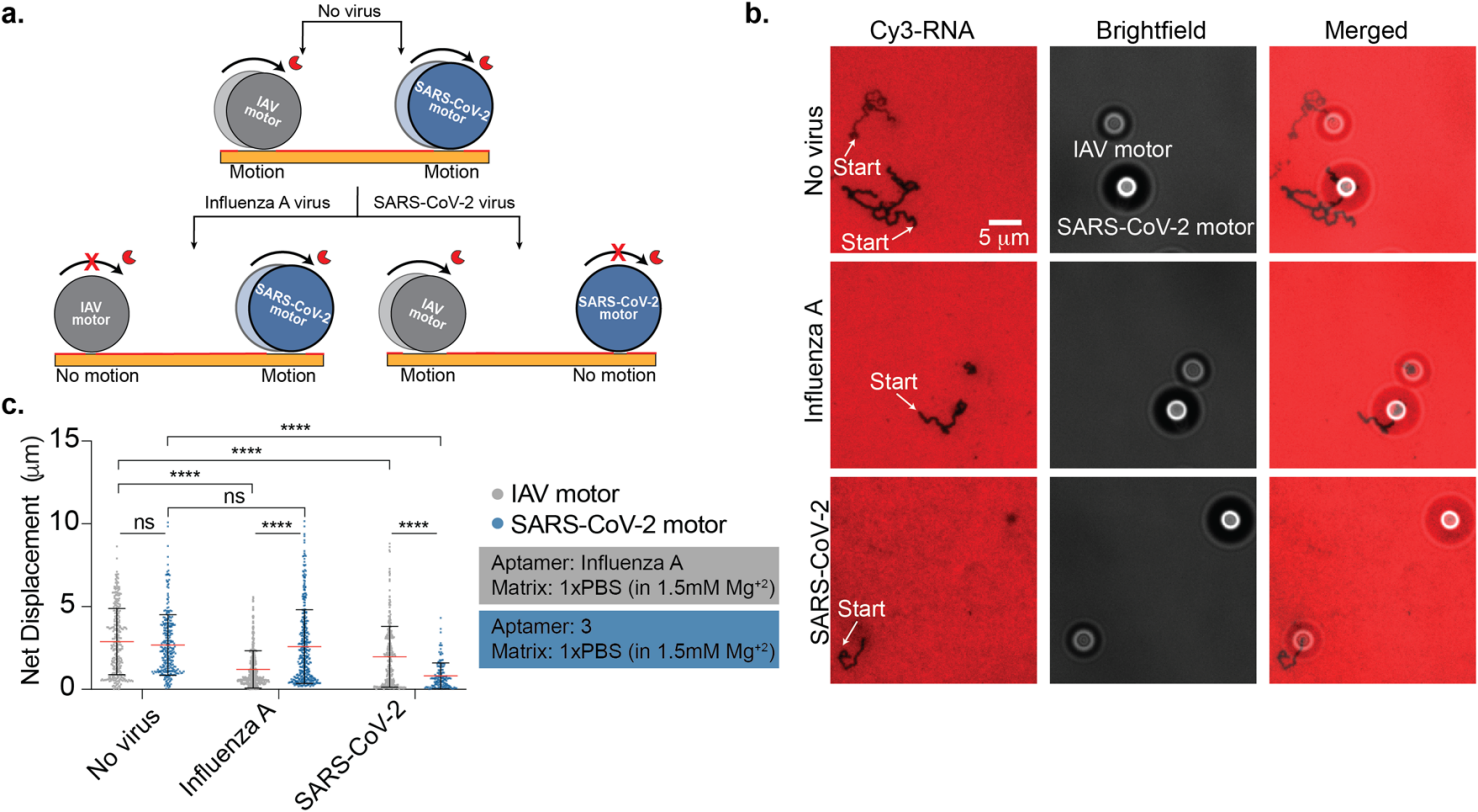
**Multiplexed detection of SARS-CoV-2 and influenza A viruses.** (a) Schematic showing multiplexed detection of influenza A virus (IAV) and SARS-CoV-2. Two types of motors specific to SARS-CoV-2 (blue, 6 μm polystyrene) and IAV (gray, 5 μm silica) were encoded based on the size and composition of the microparticles and used to simultaneously detect these two respiratory viruses. The two types of motors were mixed together and incubated for 30 min with the virus sample. (b) Fluorescence and bright-field imaging of DNA motors with no virus, 10^7^ copies/mL UV-inactivated SARS-CoV-2 WA-1, and 10^10^ copies/mL IAV. Representative images showing the two different DNA motors are shown, and each type of motor can be identified based on the bright-field particle size and contrast. Samples with SARS-CoV-2 show stalled 6 μm motors, while the IAV samples showed only stalled 5 μm particles. Samples lacking any virus showed motion of both types of motors. (c) Plots showing the net displacement for over 300 motors incubated with 10^7^ copies/mL UV-inactivated SARS-CoV-2 WA-1 and 10^10^ copies/mL IAV spiked in 1× PBS supplemented with 1.5 mM Mg^2+^. Experiments were performed in triplicate. The error bars and the red lines represent the standard deviation and the mean of the distribution, respectively. ns and **** indicate not statistically significant and *P* < 0.0001, respectively.

After validating that Rolosense is modular and can be programmed to detect other viruses, we wanted to show multiplexed detection of SARS-CoV-2 and influenza A in the same pot. To achieve this, we used two different motors: a 5 μm silica bead functionalized with influenza A aptamer and a 6 μm polystyrene bead functionalized with aptamer 3. Here we used the size and refractive index of different particles to optically encode each motor in a label-free manner using bright-field contrast.^22^ The chip was functionalized with 25% influenza A aptamer and 25% aptamer 3. As depicted in Figure 4a, when the influenza A motors (5 μm silica) and SARS-CoV-2 motors (6 μm polystyrene) were not incubated with virus, they responded with motion in the presence of RNaseH. We observed long depletion tracks in the Cy3 channel for both motors, and analysis from bright-field particle tracking of over 300 motors resulted in net displacements of 2.88 ± 2.00 μm and 2.68 ± 1.83 μm for the influenza A and SARS-CoV-2 motors, respectively (Figure 4b,c and Supplementary Movie 1). In the same tube, both motors were then incubated with 10^10^ copies/mL influenza A virus (in 1× PBS with 1.5 mM Mg^2+^) for 30 min at room temperature. As a result, the influenza A motors remained stalled on the chip while the SARS-CoV-2 motors were free to move in the presence of RNaseH. We did not observe depletion tracks in the Cy3 channel for the influenza A motor, but the SARS-CoV-2 motors formed long depletion tracks. Bright-field particle tracking confirmed this result, as the net displacement of the influenza A virus decreased compared to no virus and the SARS-CoV-2 motors exhibited an average net displacement of 2.60 ± 2.23 μm (Figure 4c and Supplementary Movie 2). The motors were also incubated with 10^7^ copies/mL SARS-CoV-2 WA-1 in 1× PBS with 1.5 mM Mg^2+^. In this condition, no tracks were observed for the SARS-CoV-2 motor, but the influenza A motors formed long tracks (Figure 4b). The average net displacement of the influenza A motors was 1.97 ± 1.84 μm, compared to 0.81 ± 0.77 μm for the SARS-CoV-2 motors (Figure 4c and Supplementary Movie 3). This slight reduction in the IAV motor motion in response to 10^7^ copies/mL SARS-CoV-2 virus is likely due to the influenza A aptamer having a small cross-reactivity to SARS-CoV-2. As a control, we also incubated the motors with both viruses, and they remained stalled on the chip (Supplementary Movie 4). All in all, using different size beads with different optical intensities, we have demonstrated the possibility of multiplexed viral detection on the same chip.

## Detecting SARS-CoV-2 via Smartphone Microscope Readout

**Figure 5:**
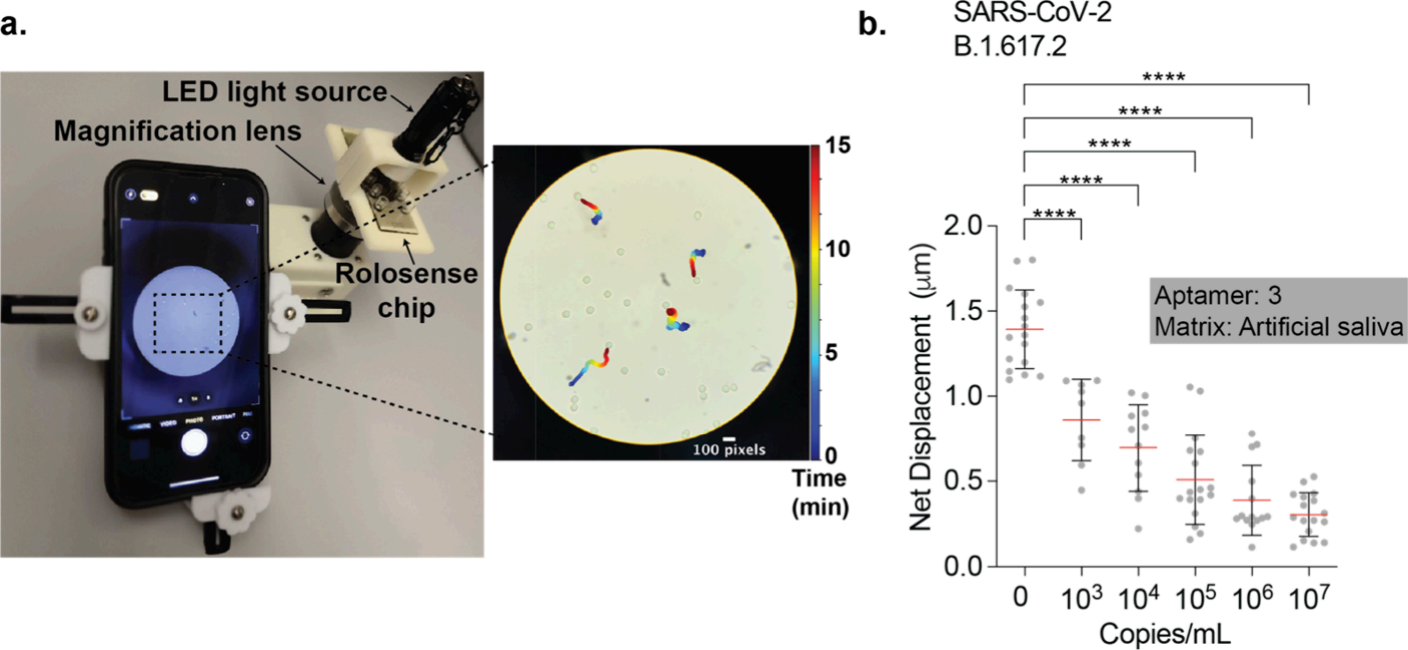
**Detecting SARS-CoV-2 B.1.617.2 using smartphone-based readout.** (a) Setup of cellphone microscope (Cellscope) which is 3D-printed and includes an LED flashlight along with a smartphone holder and simple optics. The representative microscopy image shows an image of DNA motors that were analyzed by using our custom particle tracking analysis software. Moving particles show a color trail that indicates position–time (0 → 15 min). The scale bar is 100 pixels (or 30 μm), and the diameter of the motors is 5 μm. (b) Plots showing net displacement for over 10 motors incubated with different concentrations of UV-inactivated SARS-CoV-2 B.1.617.2 samples spiked in artificial saliva. The net displacement of the motors was calculated from 15 min videos acquired using a cellphone camera. The error bars and the red lines represent the standard deviation and the mean of the distribution, respectively. The motors were functionalized with aptamer 3, and experiments were run in triplicate. **** indicates *P* < 0.0001.

Smartphone-based sensors have captured the interest of the public health community because of their global ubiquity and their ability to provide real-time geographical information on infections.^37^ Rolosense is highly amenable to smartphone readout because smartphone cameras modified with an external lens can easily detect the motion of micron-sized motors. As a proof of concept, we used a smartphone (iPhone 13) to detect the motion of Rolosense motors exposed to artificial saliva spiked with SARS-CoV-2. We used a simple smartphone microscope setup (Cellscope) as shown in Figure 5a, which includes an LED light source and 10× magnification lens. For these experiments, we functionalized DNA motors and chip with aptamer 3. SARS-CoV-2 B.1.617.2 stocks were serially diluted with artificial saliva. The DNA motors were added to these known concentrations of virus, and the samples were incubated for 30 min at room temperature. Following incubation in artificial saliva, the samples were added to the Rolosense chip and imaged for motion via smartphone. The smartphone-analyzed time-lapse imaging data agreed with that of high-end microscopy analysis, and we found that in 15 min time-lapse videos we could detect the presence of SARS-CoV-2 in artificial saliva with an LoD of ∼10^3^ copies/mL (Figure 5b and Supplementary Movies 5 and 6). Note that the net displacements and standard deviations are smaller than the data recorded with the high-end optical microscope due to the shorter readout time and that the smartphone assay does not have a perfect focus system so only the motors that meet certain criteria are analyzed (e.g., those that are fully in focus over time), while the high-end microscope assesses all motors. Nevertheless, our results show the feasibility of label-free SARS-CoV-2 sensing using a smartphone camera.

## Detecting SARS-CoV-2 in Breath Condensate Generated Samples

**Figure 6:**
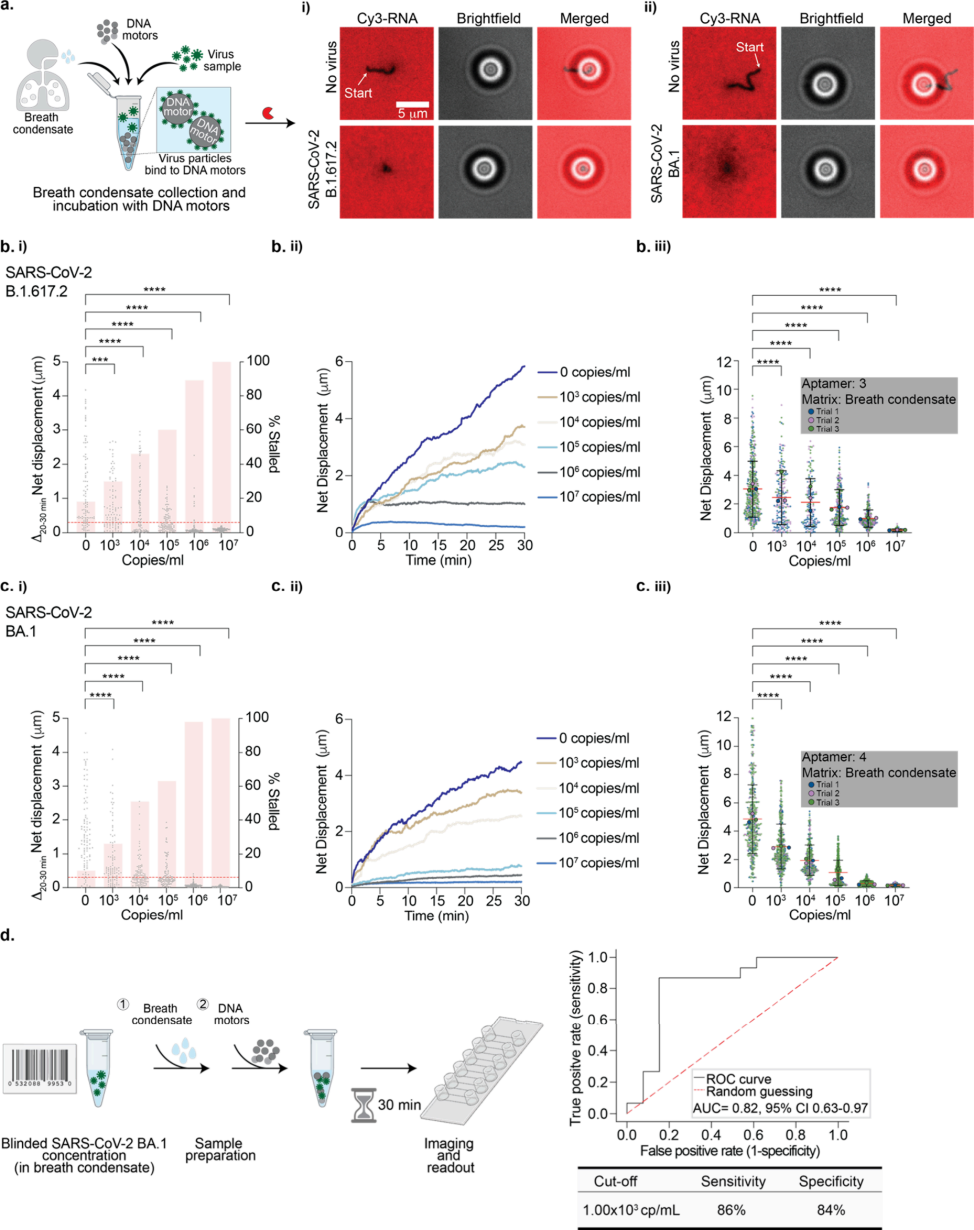
**Detecting SARS-CoV-2 virus in breath condensate.** (a) (left) Schematic of breath condensate sample collection and incubation of DNA-based motors with spiked-in virus particles. (i) Fluorescence and bright-field imaging of aptamer-3-modified DNA-based motors without virus and with 10^7^ copies/mL SARS-CoV-2 B.1.617.2. (ii) Fluorescence and bright-field imaging of aptamer-4-modified DNA motors without virus and with 10^7^ copies/mL SARS-CoV-2 BA.1. Samples without virus show long depletion tracks in the Cy3-RNA channel, but no tracks are observed following sample incubation with 10^7^ copies/mL SARS-CoV-2 B.1.617.2 and BA.1. (b) (i) Plots of the Δnet displacement as well as the percentage of motors stalled in the last 10 min of the 30 min time-lapse for 100 motors incubated with different concentrations of SARS-CoV-2 B.1.617.2. A threshold of 0.300 μm (red dotted line) was used for the percentage of stalled motors. (ii) Plots of the net displacement over the 30 min time-lapse for 30 motors incubated with different concentrations of SARS-CoV-2 B.1.617.2. (iii) Superplots of net displacement of over 300 motors for three independent replicates. Each motor is color-coded based on the triplicate data: blue for trial 1, pink for trial 2, and green for trial 3. The mean for each trial is superimposed on top of the plots. The error bars and the red lines represent the standard deviation and the mean of the distribution, respectively. (c) (i) Plots of the Δnet displacement as well as the percentage of motors stalled in the last 10 min of the 30 min time-lapse for 100 motors incubated with different concentrations of SARS-CoV-2 BA.1. To calculate a percentage of stalled motors, a 0.300 μm threshold was used (red dotted line). (ii) Plots of the net displacement over the 30 min time-lapse for 30 motors incubated with different concentrations of SARS-CoV-2 BA.1. (iii) Superplots of net displacement of over 300 motors for three independent replicates. Each motor is color-coded based on the triplicate data: blue for trial 1, pink for trial 2, and green for trial 3. The mean for each trial is superimposed on top of the plots. The error bars and the red lines represent the standard deviation and the mean of the distribution, respectively. Both SARS-CoV-2 B.1.617.2 and BA.1 were UV-inactivated. **** indicates *P* < 0.0001. (d) (left) Schematic of the blinded LoD challenge panel in which blinded SARS-CoV-2 BA.1 samples were diluted in breath condensate and incubated with aptamer-4-modified DNA-based motors. After 30 min, the motors were added to the Rolosense chip modified with aptamer 4 and imaged. (right) Receiver operating characteristic (ROC) curve evaluating the discrimination between SARS-CoV-2 BA.1-positive and -negative samples. The area under the curve is 0.82, and the 95% confidence interval (CI) is 0.63–0.97. The sensitivity and specificity values are 86% and 84%, respectively, with the best cutoff value at 1.00 × 10^3^ copies/mL.

We aimed to better predict assay performance under real-world conditions by using exhaled breath condensate as the sensing medium since exhaled breath offers the most noninvasive and accessible biological markers for diagnosis. Exhaled breath is cooled and condensed into a liquid phase and consists of water-soluble volatiles as well as nonvolatile compounds.^38^ Breath condensate has already been used as a sampling medium for breath analysis for detection of analytes such as viruses, bacteria, proteins, and fatty acids.^39^ We first collected breath condensate and mixed it with our motors without any virus to test whether Rolosense can tolerate breath condensate as the medium (Figure 6a). We used a commercial breath condensate collection tube (Figure S18a). Our results indicate that breath condensate did not affect the robustness of our assay, as motors without virus displayed net displacements comparable to those of motors diluted in 1× PBS (Figure S18b). Moreover, we also found that the breath condensate displayed little DNase and RNase activity, as indicated by the high fluorescence signal of the RNA monolayer, which is well suited for Rolosense.

To determine the LoD of SARS-CoV-2 sensing in the breath condensate, we prepared samples in a similar manner as in artificial saliva. We first functionalized the DNA-based motors and chip with aptamer 3. B.1.617.2 stocks were serially diluted in the collected breath condensate. The DNA-based motors were incubated with the virus samples in breath condensate for 30 min at room temperature. After incubation, the samples were added to the Rolosense chip and imaged. Again, we analyzed and compared the fraction of stalled motors as well as the change in net displacement in the final 10 min of our time-lapse as a function of virus concentration. From bright-field particle tracking, we observe that in the final 10 min of the time-lapse 100% of the motors are stalled when incubated with 10^7^ copies/mL B.1.617., compared to only 18% of motors without virus (Figure 6b(i)). The Δnet displacement in the *t* = 20–30 min window of the time-lapse for *N* = 100 motors incubated with 10^7^ copies/mL virus was 0.09 ± 0.04 μm. In contrast, the Δnet displacement (*t* = 20–30) for motors without any virus was 1.24 ± 1.13 μm. These findings were also clear in the net displacement over time for *N* = 30 motors, in which the net displacement decreased with increasing virus concentration (Figure 6b(ii)). From triplicate data sets, we demonstrated an LoD of 10^3^ copies/mL for SARS-CoV-2 B.1.617.2 (Figure 6b(iii)). Our results show that the LoD is maintained when the breath condensate is used as the virus-sensing matrix. Over the course of this study, we became aware of a “universal” aptamer aimed at targeting the S1 subunit of the spike protein of the BA.1 variant with high affinity (*K*_D_ = 5 nM).^40^ Therefore, to increase sensitivity in detecting the SARS-CoV-2 BA.1 variant, we functionalized our motor and chip surfaces with this BA.1-specific aptamer. As shown in Figure 6c, our data suggest that with this new aptamer we can detect the BA.1 variant at concentrations as low as 10^3^ copies/mL and possibly as low as 10^2^ copies/mL. These LoDs are highly promising, as recent studies indicate that at early stages of infection with SARS-CoV-2 the estimated breath emission rate is 10^5^ virus particles/min, which suggests that 1 min of breath condensate collection will provide sufficient material for accurate SARS-CoV-2 detection.^41^

To validate the sensitivity and specificity of our assay, we tested a blinded LoD challenge panel for SARS-CoV-2 BA.1. The blinded samples were prepared in breath condensate, and only a unique identification code was provided for each sample (Figure 6d). The DNA-based motors and chips were functionalized with aptamer 4, as it demonstrated the best performance with BA.1. The DNA-based motors were incubated with the blinded virus samples in breath condensate for 30 min at room temperature. After incubation, the samples were added to the Rolosense chip and imaged. Each of the blinded samples had varying net displacements and were thus normalized to the no-virus control for each chip surface (Figure S19). Moreover, the exhaled breath condensate used for the virus sample dilutions was not identical to the exhaled breath condensate that was used for the no-virus controls, and this resulted in the surprising situation where some samples displayed motion that was greater than our negative (no virus) controls. Accordingly, we normalized each unknown sample to a control well run on the same chip. This further controlled for variations in surface quality and other batch-to-batch differences. The samples were then assigned concentration values as well as a positive or negative call assuming a threshold of 10^3^ copies/mL. When the samples were unblinded, there were two false positives and two false negatives out of the 28 samples tested. A possible explanation for these inaccuracies could be that they are due to multiple freeze/thaw cycles the samples endured during preparation, which would lead to a decrease in protein stability over time.^42,43^ Nonetheless, as shown in Figure 6d, our conservative measures of sensitivity and specificity, using a cutoff of 1 × 10^3^ copies/mL, were 86% and 84%, respectively.

## Conclusions

We developed a mechanically based detection method of SARS-CoV-2 viral particles that is label-free and does not require fluorescence readout or absorbance measurements. Because the motor detects the virus itself rather than the nucleic acid material, there is no need for enzymatic amplification or sample processing steps. One striking feature of Rolosense is that it introduces a new concept in biosensor design that employs a “chemical-to-mechanical transduction” mechanism based on performing a mechanical test of the analyte, and the outcome of this mechanical test is the conversion of viral binding into motion output. The motors only stall if the mechanical stability of virus-binding ligands, which in our case are aptamers, exceeds the forces generated by the motor. The aptamer–spike protein rupture force is the fundamental parameter we measure, rather than the *K*_D_ of the aptamer. An additional potential advantage to mechanical transduction is that it may reduce nonspecific binding and detect transient interactions. While force spectroscopy has yet to be performed on aptamer–spike complexes, ACE2–spike complexes with similar affinity do show rupture forces of 57 pN when using 800 pN/s loading rates.^44^ Our past work estimated that each motor generates ∼100 pN of force,^28^ but likely in our case, this force is dampened because we have significantly lower density of DNA and RNA on the motor and chip, respectively. Indeed, our recent modeling^45^ suggests that lowering the magnitude of force generated by the motor can lead to enhanced biosensor performance. These estimates suggest that a single virus particle presenting 20–40 copies of trimeric spike protein^46^ will lead to motor stalling. Interestingly, when we used GFP-tagged VLPs in Rolosense we found that a population of stalled motors colocalized with single VLPs (Figure S20). Taken together, these results suggest that the Rolosense motor can respond to and report on single SARS-CoV-2 virions.

We demonstrated that in artificial saliva we can detect concentrations down to 10^3^ copies/mL SARS-CoV-2 B.1.617.2, WA-1, and the variant of concern BA.1. This limit of detection is clinically relevant, as in the early stages of infection, especially when patients are symptomatic, the viral load in respiratory secretions can be quite high, often exceeding 10^5^ viral copies/mL. While the detection of 10^3^ viral copies is lower than the typical viral load found in symptomatic individuals, it ensures a robust diagnostic capability for the assay, even in cases where sample dilution or degradation might result in lower viral concentrations. To further validate the performance of our assay, we tested for cross-reactivity with other respiratory viruses, such as the seasonal common cold viruses HCoV OC43 and 229E as well as influenza A. We did not observe a distinguishable effect on the Rolosense response. A key advantage of Rolosense is its ability to multiplex and detect multiple respiratory viruses in the same assay. This capability is important for point-of-care diagnostics and in minimizing false-positive results due to similar symptoms caused by other respiratory viruses. We show that by encoding different virus-specific DNA motors through size and refractive index we can distinguish between SARS-CoV-2 and influenza A in one pot. With the aim of enabling the key steps for a point-of-care diagnostic, we demonstrated that the Rolosense motors and chip can be used to conveniently detect SARS-CoV-2 using a smartphone and a magnifying lens as the reader. The assay was performed using a rapid ∼15 min readout without any intervention. Our assay is also suitable for exhaled breath condensate testing, as we demonstrated an LoD of 10^3^ copies/mL for the B.1.617.2 and BA.1 variants with a sensitivity and specificity of 86% and 84%, respectively. While these values are below those of many of the recent FDA-approved diagnostics, our work represents the initial report of a new technology that still requires optimization in future iterations, which will certainly boost these levels.

Our reported LoD of 10^3^ copies/mL is better than that of lateral flow assays like the BinaxNOW COVID-19 Ag Card (Abbott Diagnostics Scarborough, Inc.) which have an LoD of 10^5^ copies/mL for the BA.1 variant.^47^ Rolosense takes advantage of multivalent binding through the arrangement of multiple aptamers on the motor and chip surface, which may contribute to an LoD that is better than that of monomeric assays like LFA. Qualitatively, we find that the LoD in our assay is influenced by the affinity of the aptamer for the SARS-CoV-2 spike protein. In our initial screens using different SARS-CoV-2 aptamers, we discovered that many aptamers do not work due to poor affinity or poor specificity. These data are not included. We also note that aptamer 3 has a tighter binding constant toward the SARS-CoV-2 B.1.617.2 variant, and this aptamer shows improved LoD against the B.1.617.2 variant compared to BA.1, as one would expect (Figure S15). While individual aptamer affinity is important, the arrangement of multiple aptamers on the silica sphere surface allows for simultaneous interactions with multiple epitopes on the spike protein, creating a cumulative effect that significantly increases the overall binding strength. This multivalent binding effect leads to an increased local concentration of the aptamers around the spike protein, which can substantially lower the LoD beyond what can be achieved by monovalent interactions alone. The cooperative binding facilitated by multivalency often results in a much lower dissociation rate, thus stabilizing the aptamer–virus complex, which is essential for the detection. Furthermore, the multivalent interaction on the chip, where the same aptamers are presented, enhances the likelihood of stalling by a sphere–virus complex due to increased binding events. This dual multivalency—both on the silica sphere and on the chip—amplifies the assay’s sensitivity, making it possible to detect even minimal amounts of the virus that might not be possible through monovalent binding alone.

Another strength of Rolosense is that it is highly modular, and any whole virion that displays many copies of a target can be detected using our assay with appropriate aptamers. While our current assay performs well with the SARS-CoV-2 virus, which has a high density of the S1 spike protein, detecting viruses with lower expression levels of antigens would require strategic adjustments. These could include the use of high-affinity and highly specific aptamers, optimized aptamer density and spatial arrangement on both the motors and chip surfaces, and signal amplification methods. Additionally, using a mixture of aptamers that bind to several different epitopes or proteins on the viral surface can increase the chances of binding, thus improving the assay’s sensitivity as we recently reported.^59^ Though such adaptations could be necessary, our platform’s fundamental design principles remain applicable across various target densities, potentially allowing for versatile virus detection with adjusted sensitivity. Furthermore, the modularity of Rolosense enables multiplexed detection of SARS-CoV-2 and influenza A, which can in principle be scaled up to include a panel of viral targets, as we could create tens of uniquely encoded motors. Such PCR panels for multiplexed detection of respiratory viruses are currently available,^48^ and hence, multiplexed Rolosense would find clinical applications, as our assay is rapid and can be conducted conveniently without the need for a dedicated PCR instrument.

When compared to the gold standard, reverse-transcription quantitative PCR (RT-qPCR), which has a sensitivity of 10^2^–10^3^ copies/mL, Rolosense presents a weaker LoD. However, it is essential to note that Rolosense quantifies intact viral particles, which is a direct indicator of infectious virus, as opposed to RT-qPCR, which quantifies viral RNA copies and may detect noninfectious or residual viral genetic material. This differentiation is crucial in the clinical context, where distinguishing between infectious and noninfectious individuals can impact isolation decisions and public health responses. Other techniques quantifying intact particles^49,50^ achieve a similar LoD to that of Rolosense with simple readout methods; however, Rolosense employs a fundamentally different mechanism of readout and measures the mechanical motion of DNA motors. This advantage allows for real-time monitoring of virus-binding events and bright-field readouts using biological media such as breath condensate. Compared to commercially available LFAs,^14,47^ which typically show LoDs of 10^4^–10^6^ copies/mL, Rolosense offers a significant improvement in sensitivity, approaching that of molecular tests. While LFAs provide rapid results at a low cost without the need for specialized equipment, their lower sensitivity can result in a higher rate of false negatives, particularly in the early stages of infection or in samples with low viral loads. In addition, LFA assays are challenging to multiplex. By using different-sized particles and thus different aptamers specific to distinct viral antigens, Rolosense can be designed to detect multiple viruses or virus strains simultaneously on the same chip.

There are a few limitations inherent to Rolosense. The first is the sensitivity to RNase and DNase contamination in the biological samples. We have documented this issue (Figure S21), and selective nuclease inhibitors that target RNase A, B, and C (but not RNaseH) showed excellent potential to minimize this issue. Note, however, that all assays that employ DNA or RNA aptamers for biological sensing also suffer from nuclease sensitivity. Nonetheless, we were excited to see that breath condensate is relatively low in nuclease matrix, and hence, this is well-suited for the Rolosense assay and for detecting respiratory virions. A second issue is the requirement of cold storage for RNaseH enzyme. However, RNaseH derived from thermophiles that are commercially available could circumvent this issue, as such enzymes will likely remain stable at room temperature, thus side-stepping the need for cold storage at −20 °C.

Another limitation is the need to employ virus-binding ligands that target spike protein (or other surface-displayed proteins). This is not a weakness in itself, but rather, this is a challenge when pursuing highly mutable targets such as SARS-CoV-2 and influenza that are under high evolutionary pressure to conceal their surface epitopes from immune recognition.^51^ This leads to frequent mutations in the spike protein, in contrast to nucleocapsid proteins that are infrequently altered.^52^ Our work with the “universal” spike protein aptamer shown in Figure 6 represents one solution to this problem. However, the specificity of universal aptamers is weaker, and hence, they are more likely to cross-react with similar spike-presenting coronaviruses. Further development and deployment of Rolosense shows potential toward a point-of-care system, which will greatly facilitate frequent on-site molecular diagnostics.

## Methods

### Materials

All oligonucleotides were purchased from Integrated DNA Technologies (IDT), stored at 4 °C (−20 °C for RNA), and used without purification. Their sequences, including functional group modifications, are listed in Supplementary Table 1. Stock solutions were made using Nanopure water (Barnstead Nanopure system, resistivity = 18.2 MΩ), herein referred to as DI water. Aminated silica beads (5 μm) were purchased from Bangs Laboratory (no. SA06N). Aminated polystyrene beads (6 μm) were purchased from Spherotech (no. AP-60-10). Artificial saliva was purchased from Fisher Scientific (no. NC1873815). Influenza A/PR/8/34 was purchased from Charles River Laboratories (no. 10100374). RNaseH was obtained from Takara Clontech (no. 2150A). RTube breath condensate collection device was purchased from Respiratory Research (nos. 1025, 3002, and 3001). Thin Au films were generated by using a home-built thermal evaporator system. All motor translocation measurements were performed in Ibidi sticky-slide VI0.4 (Ibidi, no. 80608) 17 mm × 3.8 mm × 0.4 mm channels. The smartphone microscope was obtained from Wilbur Lam, Emory University (10×, 0.25 NA objective and 20× WF eyepiece) (https://cellscope.berkeley.edu/).

### Microscopy

Bright-field and fluorescence images were acquired on a fully automated Nikon Inverted Research Microscope Eclipse Ti2-E with the Elements software package (Nikon), an automated scanning stage, a 1.49 NA CFI Apo TIRF 100× objective, a 0.50 NA CFI60 Plan Fluor 20× objective, a Prime 95B 25 mm scientific complementary metal–oxide–semiconductor (sCMOS) camera for image capture at 16-bit depth, a SOLA SE II 365 Light Engine for solid-state white-light excitation source, and a perfect focus system used to minimize drift during time-lapse. Bright-field time-lapse imaging was done using a 20×, 0.50 NA objective with 5 s per frame rate and an exposure time of 100 ms. Fluorescence images of Cy3 and GFP were collected using a TRITC filter set (Chroma, no. 96321) and an EGFP/FITC/Cy2/Alexa Fluor 488 filter set (Chroma, no. 96226) with an exposure time of 100 ms. All imaging was conducted at room temperature.

### Viruses

UV-inactivated SARS-CoV-2 and human corona (229E, OC43) virus samples at measured and confirmed concentrations (via ddPCR) were provided by the NIH RADx-Radical Data Coordination Center (RADx-rad DCC) at the University of California, San Diego and BEI Resources. Specifically, UV-inactivated SARS-CoV-2 isolate hCoV-19/USA/PHC658/2021 (lineage B.1.617.2; Delta variant), NR-55611, was contributed by Dr. Richard Webby and Dr. Anami Patel. The following reagent was obtained from UC San Diego: SARS-related coronavirus 2, isolate hCoV-19/USA/CA-SEARCH-59467/2021 (lineage BA.1; Omicron variant), contributed by Dr. Alex Clark, Dr. Aaron Carlin, and the UC San Diego CALM and EXCITE laboratories. This reagent was produced with help from the San Diego Center for AIDS Research (SD CFAR), an NIH-funded program (P30 AI036214), which is supported by the following NIH institutes and centers: NIAID, NCI, NHLBI, NIA, NICHD, NIDA, NIDCR, NIDDK, NIGMS, NIMH, NIMHD, FIC, and OAR. We thank RADx-rad DCC at the University of California San Diego, which is funded under NIH Grant 1U24LM013755-01. Virus samples used in this study underwent at least one freeze–thaw cycle.

### Cells and Plasmids

The human embryonic kidney HEK293T/17 cell line was obtained from ATCC (Manassas, VA, USA). Cells were grown in high-glucose Dulbecco’s modified Eagle’s medium (DMEM) (Mediatech, Manassas, VA, USA), 10% fetal bovine serum (FBS) (Sigma, St. Louis, MO, USA), 100 units/mL penicillin–streptomycin (Gemini Bio-Products, Sacramento, CA, USA), and 0.5 mg/mL G418 sulfate (Mediatech). pCAGGS-SARS-CoV-2 S D614G (no. 156421) and pcDNA3.1 vectors were obtained from Addgene (Watertown, MA, USA) and Invitrogen (Waltham, MA), respectively. HIV Gag-eGFP expression vector was a gift from Dr. Marilyn D. Resh (Memorial Sloan-Kettering Cancer Center, New York).^53^

### VLP Production and Characterization

To produce VLPs, HEK 293T/17 cells were grown to 70–80% confluency in a 100 mm plate and transfected with 4 μg of SARS-CoV-2 S D614G Env and 6 μg of HIV-1 Gag-eGFP using JetPrime transfection reagent (Polyplus-transfection, Illkirch, France). For producing bald VLPs, the SARS-CoV-2 S D614G expression vector was replaced with pcDNA3.1 empty vector. Twelve hours post-transfection, the medium was exchanged with DMEM/10% FBS supplemented with 100 units/mL penicillin–streptomycin. At 48 h post-transfection, supernatant was collected, filtered through a 0.45 μm filter, and precipitated with a Lenti-X concentrator (Clontech) at 4 °C for 12 h. The sample was centrifuged at 4 °C, 1500*g* for 45 min, and the viral pellet was resuspended in 1/100 volume of PBS, aliquoted, and stored at −80 °C. The number of particles was estimated based on the p24/Gag amount measured by enzyme-linked immunosorbent assay (ELISA) as previously described.^54^

### Quantification and Imaging of VLPs Using Single-Particle Microscopy Imaging

A no. 1.5H glass slide (25 mm × 75 mm) was cleaned by sonication in DI water for 15 min. The sample was then sonicated in 200 proof ethanol for 15 min and dried under a stream of N_2_. The glass slide was etched by piranha solution (3:7 v/v hydrogen peroxide/sulfuric acid) for 30 min to remove residual organic materials and activate hydroxyl groups on the glass (***caution**: piranha is extremely corrosive and may explode if exposed to organics*). The cleaned substrates were rinsed with DI water in a 200 mL beaker six times and washed with ethanol three times. Slides were then transferred to a 200 mL beaker containing 2% v/v APTES in ethanol for 1 h, washed with ethanol three times, and thermally cured in the oven (110 °C) for 10 min. The slides were then mounted to six-channel microfluidic cells (Sticky-Slide VI 0.4, Ibidi). A 1000× dilution of the GFP-tagged spike and bald VLP samples was created in 1× PBS and added to the APTES surface. High-resolution epifluorescence images (×100) of the GFP-tagged VLPs were acquired and used for further analysis.

### Thermal Evaporation of Gold Films

A no. 1.5H glass coverslip (25 mm × 75 mm) (Ibidi, no. 10812) was cleaned by sonication in DI water for 5 min. The sample was then subjected to a second sonication in fresh DI water for 5 min. Finally, the slide was sonicated in 200 proof ethanol (Fisher Scientific, no. 04-355-223) for 5 min and was subsequently dried under a stream of N_2_. The cleaned glass coverslip was then mounted into a home-built thermal evaporator chamber, in which the pressure was reduced to 50 × 10^–3^ Torr. The chamber was purged with N_2_ three times, and the pressure was reduced to 1–2 × 10^–7^ Torr using a turbo pump with a liquid N_2_ trap. Once the desired pressure was achieved, a 3 nm film of Cr was deposited onto the slide at a rate of 0.2 Å s^–1^, which was determined by a quartz crystal microbalance. After the Cr adhesive layer had been deposited, 5 nm of Au was deposited at a rate of 0.4 Å s^–1^. The Au-coated samples were used within 1 week of deposition.

### Fabrication of RNA/DNA Aptamer Monolayers

An Ibidi Sticky-Slide VI 0.4 flow chamber was adhered to the Au-coated slide to produce six channels (17 mm × 3.8 mm × 0.4 mm dimensions). Prior to surface functionalization, each channel was rinsed with ∼5 mL of DI water. Next, thiol-modified DNA anchor strands were added to each of the channels with 50 μL of solution of 1 μM DNA anchor in a 1 M potassium phosphate monobasic (KH_2_PO_4_) buffer. The gold film was sealed by Parafilm to prevent evaporation, and the reaction took place overnight at room temperature. After incubation, excess DNA was removed from the channel using an ∼5 mL DI water rinse. To block any bare gold sites and maximize the hybridization of RNA and DNA aptamer to the DNA anchoring strand, the surface was back-filled with 100 μL of a 100 μM solution of (11-mercaptoundecyl)hexa(ethylene glycol) (SH-PEG) (Sigma-Aldrich, no. 675105) solution in ethanol for 6 h. Excess SH-PEG was removed by an ∼5 mL rinse with ethanol and another ∼5 mL rinse with water. For a 50% RNA and 50% DNA aptamer surface, the RNA/DNA chimera (50 nM) and the surface aptamer (50 nM) were mixed and added to the surface through hybridization to the DNA anchor in 1× PBS for 12 h. For the multiplexed detection of SARS-CoV-2 and influenza A experiments, RNA/DNA chimera (50 nM), surface aptamer 3 (25 nM), and influenza A surface aptamer (25 nM) were mixed and added to the surface through hybridization to the DNA anchor in 1× PBS for 12 h. The wells were again sealed with Parafilm to prevent evaporation, and the resulting RNA monolayer remained stable for days.

### Synthesis of Azide-Functionalized Motors

Before functionalization with azide, the silica and polystyrene beads were washed to remove any impurities. For the wash, aminated silica beads (1 mg) were centrifuged down for 5 min at 15,000 rpm in 1 mL of DI water. Similarly, aminated polystyrene beads (1 mg) were centrifuged down for 10 min at 15,000 rpm in 1 mL of DI water with 0.005% surfactant (Triton-X). The supernatant was discarded, and the resulting particles were resuspended in 1 mL of DI water (silica beads) and 1 mL of DI water with 0.005% Triton-X (polystyrene beads). This was repeated three times, and the supernatant was discarded after the final wash. Azide-functionalized particles were then synthesized by mixing 1 mg of aminated silica and polystyrene beads with 1 mg of azido acetic NHS ester (BroadPharm, no. BP-22467). This mixture was subsequently diluted in 100 μL of dimethyl sulfoxide (DMSO) and 1 μL of a 10× diluted triethylamine stock solution in DMSO. The reaction proceeded overnight for 24 h at room temperature, and the azide-modified silica particles were purified by adding 1 mL of DI water and centrifuging down the particles at 15,000 rpm for 5 min. The azide-modified polystyrene particles were purified in a similar manner except that they were centrifuged for 10 min in 0.005% Triton-X. The supernatant was discarded, and the resulting particles were resuspended in 1 mL of DI water. This process was repeated seven times, and during the final centrifugation step, the particles were resuspended in 100 μL of DI water to yield an azide-modified particle stock. The azide-modified particles were stored at 4 °C in the dark and were used within 1 month of preparation.

### Synthesis of High-Density DNA Silica and Polystyrene Motors

High-density DNA-functionalized motors were synthesized by adding a total of 5 nmol (in 5 μL) of alkyne-modified DNA stock solution to 5 μL of azide-functionalized motors. For motors with 10% aptamer, 4.5 nmol of DNA leg and 0.5 nmol of particle aptamer 1, 2, 3, 4, or influenza A particle aptamer were mixed with 5 μL of azide-functionalized particles. The particles and DNA were diluted with 25 μL of DMSO and 5 μL of 2 M triethylammonium acetate buffer (TEAA). Next, 4 μL of a supersaturated stock solution of ascorbic acid was added to the reaction as a reducing agent. Cycloaddition between the alkyne-modified DNA and azide-functionalized particles was initiated by adding 2 μL from a 10 mM copper tris((1-benzyl-1*H*-1,2,3-triazol-4-yl)methyl)amine (Cu-TBTA) stock solution in 55 vol % DMSO (Lumiprobe, no. 21050). Note that since the aptamer strands on the motors are anchored using the same click reaction as that used for the DNA legs, we assume that the solution percent of the aptamer reflects the surface density percent of the aptamer. The reaction was incubated for 24 h at room temperature on a shaker, and the resulting DNA-functionalized motors were purified by centrifugation. The motors were centrifuged at 15,000 rpm for 10 min, after which the supernatant was discarded and the motors were resuspended in 1 mL of a 1× PBS and 10% w/v Triton-X solution. This process was repeated seven times, with the motors resuspended in 1 mL of 1× PBS only for the fourth to sixth centrifugations. During the final centrifugation, the motors were resuspended in 50 μL of 1× PBS. The high-density DNA-functionalized motors were stored at 4 °C and protected from light.

### Preparation of Antibody-Coated Motors and Chips

To prepare DNA–antibody conjugates, 25 μg of monoclonal rabbit S1 (Genetex, no. GTX635654) and monoclonal mouse S2 (no. GTX632604) in 70 μL of 1× PBS was mixed with 80 mM succinimidyl 4-(*N*-maleimidomethyl)cyclohexane-1-carboxylate (SMCC) in 4 μL of dimethylformamide (DMF). The solution was incubated on ice for 2 h. Excess SMCC was removed from the maleimide-functionalized antibodies using Zeba spin columns (7000 MWCO, eluent: 1× PBS). Thiol-modified DNA oligomers (50 nmol) were reduced using dithiothreitol (DTT) (200 mM) for 2 h at room temperature. The reduced DNA oligomers were purified using NAP-5 columns (GE Healthcare). Deionized water was used as the eluent. Then the reduced DNA was mixed with the maleimide-functionalized antibodies in 1× PBS overnight at 4 °C. DNA–antibody conjugates were purified and concentrated using Amicon Ultra centrifugal filters (100 kDa MWCO). The DNA–antibody conjugates were then added to the DNA-based motors and chips via hybridization.

### Breath Condensate Collection

Breath condensate was collected using the R tube breath condensate collection device from Respiratory Research (nos. 1025, 3002, and 3001). The R tube breath condensate collection device consists of three parts: a disposable R tube collector, a cooling sleeve, and the plunger. First, the cooling sleeve was placed in a −20 °C freezer for 15 min. After 15 min, the cooling sleeve was placed on top of the disposable R tube collector, and exhaled breath condensate was collected by breathing into the mouthpiece of the R tube collector for 2–5 min. The vapor emerging from the breath was condensed onto the sides of the R tube collector. Following 2–5 min of breathing into the R tube collector, the mouthpiece was removed from the bottom of the R tube collector, and the tube was placed on top of the plunger and pushed through it. The exhaled breath condensate was collected into a pool of liquid at the top. The condensed breath was then transferred into an Eppendorf tube and used in creating serial dilutions of the virus samples.

### Motor Translocation

Before beginning the experiments, known concentrations of virus samples were serially diluted in either 1× PBS, artificial saliva, or collected breath condensate to create samples of different virus concentrations. The DNA-based motors were then incubated with different concentrations of virus samples for 30 min at room temperature. This was done by adding 1 μL of DNA-based motors (∼800 particles/μL) in 49 μL of either 1× PBS (±virus particles) or a matrix such as artificial saliva or breath condensate (±virus particles). After 30 min of incubation, the DNA-based motors were added to the Rolosense chip, which was prewashed with 5 mL of 1× PBS to remove excess unbound RNA and surface aptamer. Motor translocation was then initiated with 100 μL rolling buffer, which consisted of 73 μL of DI water (73%), 5 μL of 10× RNaseH reaction buffer (25 mM Tris, pH 8.0, 8 mM NaCl, 37.5 mM KCl, 1.5 mM MgCl_2_), 10 μL of formamide (10%), 10 μL of 7.5% (g mL^–1^) Triton X (0.75%), 1 μL of RNaseH in 1× PBS (5 units), and 1 μL of 1 mM DTT (10 μM). RNaseH enzyme stock was stored on ice for up to 2 h. Particle tracking was achieved through bright-field imaging by recording a time-lapse at 5 s intervals for 30 min via the Nikon Elements software. High-resolution epifluorescence images (×100) of fluorescence-depletion tracks as well as VLP fluorescence intensity were acquired to verify that particle motion resulted from processive RNA hydrolysis and confirm VLP binding. The resulting time-lapse files and high-resolution epifluorescence images were then saved for further analysis. The LoD was determined from the lowest concentration of sample incubated with virus that was significantly higher than that of the negative control.

### Preparation and Testing of Blinded LoD Challenge Panel

A panel of 28 SARS-CoV-2 BA.1 samples was prepared in exhaled breath condensate and provided by the DCC core supported by the NIH RADx-rad effort. The panel included four blank samples (no virus, only exhaled breath condensate) and 24 samples in concentrations ranging from 1.00 × 10^2^ to 1.02 × 10^5^ copies/mL in triplicate. The virus samples were isolated from cell culture media and were then diluted into an exhaled breath condensate. The samples were blinded, and only a unique tracking number was provided for each sample. To test the blinded SARS-CoV-2 BA.1 samples, the DNA-based motors and chip were modified with aptamer 4. The aptamer-functionalized motors (1 μL) were first incubated with each sample (50 μL) for 30 min at room temperature. For every Rolosense chip, a control with no virus (1 μL of DNA-based motors and 50 μL of breath condensate) was tested, in addition to the blinded virus samples. After the 30 min of incubation at room temperature, the samples were added to the Rolosense chip, and motor translocation was initiated as described above (100 μL of rolling buffer: 73 μL DI water (73%), 5 μL of 10× RNaseH reaction buffer (25 mM Tris, pH 8.0, 8 mM NaCl, 37.5 mM KCl, 1.5 mM MgCl_2_), 10 μL of formamide (10%), 10 μL of 7.5% (g mL^–1^) Triton X (0.75%), 1 μL of RNaseH in 1 × PBS (5 units), and 1 μL of 1 mM DTT (10 μM)). After the 28 samples were tested, concentration values as well as “±” virus notations were assigned to each sample based on the net displacement value normalized to the no-virus control. The samples were then unblinded in an online platform prepared by the team at RADx-rad that had the correct or true virus concentrations. The receiver operating characteristic (ROC) curve was constructed using the true labels for the samples as well as the true positive and false positive rates.

### Image Processing and Particle Tracking

Image processing and particle tracking were performed in Fiji (ImageJ) as well as Python. The time-lapse app *Lapse It* v. 5.02 was used to record time-lapse videos (5 s/frame) of DNA motors on a smartphone. The bioformats toolbox in Fiji (ImageJ) enabled direct transfer of Nikon Elements image files (*.nd2) into the Fiji (ImageJ) environment, where all image/video processing was performed. Particle tracking was performed using the 2D/3D particle tracker from the Mosaic plugin in Fiji (ImageJ),^55^ in which we generated .csv files with particle trajectories that were used for further analysis. The algorithms for processing the data for motor trajectories, net displacements, and speeds were performed on Python v3.7.4. Calculation of drift correction was adapted from trackpy (https://soft-matter.github.io/trackpy). The full Python script from bright-field acquisition data can be found at https://github.com/spiranej/particle_tracking_. One-way ANOVA and two-way ANOVA (Figure 4c) statistical analyses were performed in GraphPad v9.1.0.

## Data Availability

Source statistical data are provided with this paper. Additional data sets generated are available from the corresponding author on reasonable request. The Python script from bright-field acquisition data regarding net displacements and particle ensemble trajectories can be found at https://github.com/spiranej/particle_tracking_

## Supporting Information Available

Supplementary Table 1, Figures S1–S21, and descriptions of the supplementary movies (PDF)
Supplementary Movie 1: Time-lapse video of particle trajectories of SARS-CoV-2 and influenza A motors that were not incubated with virus (AVI)
Supplementary Movie 2: Time-lapse video of particle trajectories of SARS-CoV-2 and influenza A motors that were incubated with only influenza A virus (AVI)
Supplementary Movie 3: Time-lapse video of particle trajectories of SARS-CoV-2 and influenza A motors that were incubated with only SARS-CoV-2 virus (AVI)
Supplementary Movie 4: Time-lapse video of particle trajectories of SARS-CoV-2 and influenza A motors that were incubated with both viruses (AVI)
Supplementary Movie 5: Time-lapse smartphone video acquired following RNaseH addition of DNA-based motors diluted in artificial saliva without virus present (AVI)
Supplementary Movie 6: Time-lapse smartphone video acquired following RNaseH addition of DNA-based motors incubated in artificial saliva spiked with SARS-CoV-2 virus (AVI)

## Supplementary Material

oc4c00312_si_001.pdf

oc4c00312_si_002.avi

oc4c00312_si_003.avi

oc4c00312_si_004.avi

oc4c00312_si_005.avi

oc4c00312_si_006.avi

oc4c00312_si_007.avi
