# Supplementary material for: Rolosense: Mechanical Detection of SARS-CoV-2 Using a DNA-Based Motor
Source: ACS Cent Sci. 2024 May 21;10(7):1332–47. doi: 10.1021/acscentsci.4c00312 (PMC11273449; doi:10.1021/acscentsci.4c00312)
Supplement: Supplementary file 1 — oc4c00312_si_001.pdf [file oc4c00312_si_001.pdf]

Supporting Information

Rolosense: Mechanical Detection of SARS-CoV-2 Using a DNA-Based Motor

Selma Piranej[1], Luona Zhang[1], Alisina Bazrafshan[1], Mariana Marin[2,3], Gregory B. Melikian[2,3], Khalid Salaita[1,4*]

[1]Department of Chemistry, Emory University, Atlanta, GA 30322 (USA). [2]Department of Pediatrics, Emory University School of Medicine, Atlanta, Georgia 30322 (USA). [3]Children's Healthcare of Atlanta, Atlanta, Georgia 30322 (USA).[4]Wallace H. Coulter Department of Biomedical Engineering, Georgia Institute of Technology and Emory University, Atlanta, GA 30322 (USA).

*Correspondence should be addressed to: k.salaita@emory.edu

Table of Contents

Supplementary Table 1: Oligonucleotide sequence design 3
Figure S1. Secondary structure prediction and alignment of aptamers. 4
Figure S2. Fluorescence imaging of Rolosense chip with aptamer. 5
Figure S3. DNA-based motors tolerate certain densities of aptamer. 6
Figure S4. Quantification of VLPs using single particle imaging. 7
Figure S5. Net displacement of aptamer-modified motors without VLPs 8
Figure S6. Using motors modified with antibodies to detect SARS-CoV-2. 9
Figure S7. DNA-based motors tolerate artificial saliva as a medium for virus sensing. . 10
Figure S8. Detecting SARS-CoV-2 WA-1 virus in artificial saliva. 11
Figure S9. DNA-based motors incubated with virus bind specifically to aptamers on the Rolosense chip. 12
Figure S10. Eliminating washing steps does not affect the integrity of Rolosense. 13
Figure S11. Decreasing DNA motor and virus incubation time does not affect the overall performance of Rolosense. 14
Figure S12. Motor trajectories over time as a function of increasing virus concentration. 15
Figure S13. Modeling motor-virus encounter rates 17
Figure S14. Detection sensitivity of SARS-CoV-2 in artificial saliva. 18
Figure S15. Detecting SARS-CoV-2 BA.1 strain in artificial saliva. 19
Figure S16. Motors demonstrate a specific response to SARS-CoV-2 viruses. 20
Figure S17. DNA motors detect influenza A virus. 21
Figure S18. DNA motors tolerate breath condensate as a virus sensing medium. 22
Figure S19. Blinded limit of detection (LoD) challenge panel. 23
Figure S20. DNA-based motors stall in the presence of a single viral particle. 25
Figure S21. Rolosense chip can tolerate saliva in the presence of RNase inhibitors. ... 26
Supplementary Movies 27
References 28

Supplementary Table 1: Oligonucleotide sequence design

ID	Sequences (5'-3')
DNA Anchor	/5AmMC6/GAGAGAGATGGGTGCTTTTTTTTTTTTTTT/3ThiolMC3-D/
RNA/DNA Chimera	GCACCCATCTCTCTCCCCCCrCrUrGrUrGrArUrUrGrArUrUrArCrU/3Cy3Sp/
DNA Leg	/5Hexynyl/TTTTTTTTTTTTTTTAGTAATCAATCACAG
Particle Aptamer 1[1]	/5Hexynyl/TTTTTCGCGGTCATTGTGCATCCTGACTGACCCTAAGGTG CGAACATCGCCCGCG
Surface Aptamer 1	GCACCCATCTCTCTCCGCGGTCATTGTGCATCCTGACTGACCCTAA GGTGCGAACATCGCCCGCG
Particle Aptamer 2[2]	/5Hexynyl/TTTTTGGGAGAGGAGGGAGATAGATATCAACCCATGGTAG GTATTGCTTGGTAGGGATAGTGGGCTTGATGTTTCGTGGATGCCACA GGAC
Surface Aptamer 2	GCACCCATCTCTCTCGGAGAGGAGGGAGATAGATATCAACCCATGGTAGGT ATTGCTTGGTAGGGATAGTGGGCTTGATGTTTCGTGGATGCCACAGGAC
Particle Aptamer 3[2]	/5Hexynyl/TTTTTCCCATGGTAGGTATTGCTTGGTAGGGATAGTGGG
Surface Aptamer 3	GCACCCATCTCTCTCCCCATGGTAGGTATTGCTTGGTAGGGATAGTGGG
Particle Aptamer 4[3]	/5Hexynyl/TTTTTACGCCAAGGTGTCACTCCGTAGGGTTTGGCTCCGGGCC TGGCGTCGGTCG CGAAGCATCT CCTTGGCGT
Surface Aptamer 4	GCACCCATCTCTCTCACGCCAAGGTGTCACTCCGTAGGGTTTGGCTCCG GGCCTG GCGTCGGTCGCGAAGCATCTCCTTGGCGT
Influenza A Particle[4] Aptamer	/5Hexynyl/TTTTTGGCAGGAAGACAAACAGCCAGCGTGACAGCGACGCG TAGGGACCGGCATCCGCGGGTGGTCTGTGGTGCTGT
Influenza A Surface Aptamer	GCACCCATCTCTCTCGGCAGGAAGACAAACAGCCAGCGTGACAGCGA CGCGTAGGGACCGGCATCCGCGGGTGGTCTGTGGTGCTGT

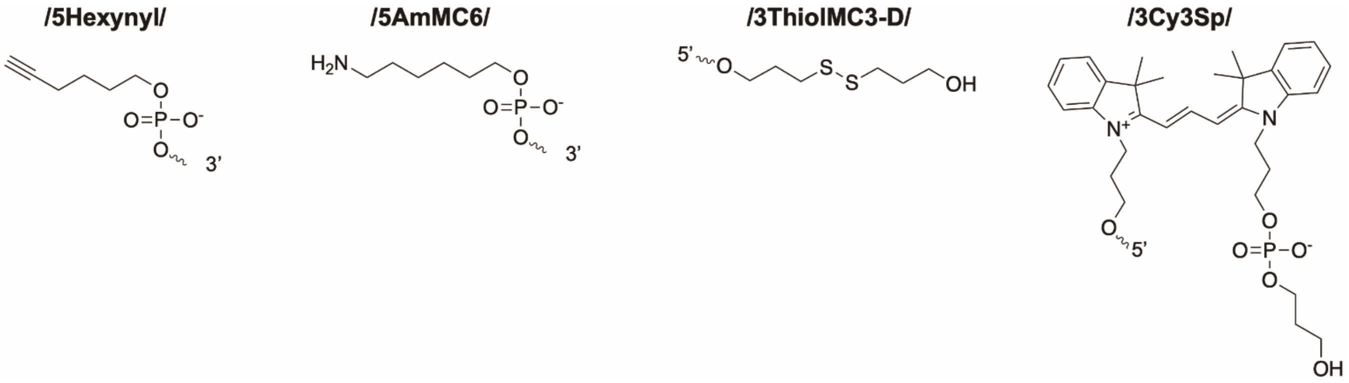

Supplementary Table 1. Table summarizing the sequences of oligonucleotides used in the design of the Rolosense assay displayed in a 5’ to 3’ orientation. The 5’ and 3’ DNA and RNA modifications are indicated in the table and illustrated below it.

Figure S1. Secondary structure prediction and alignment of aptamers.

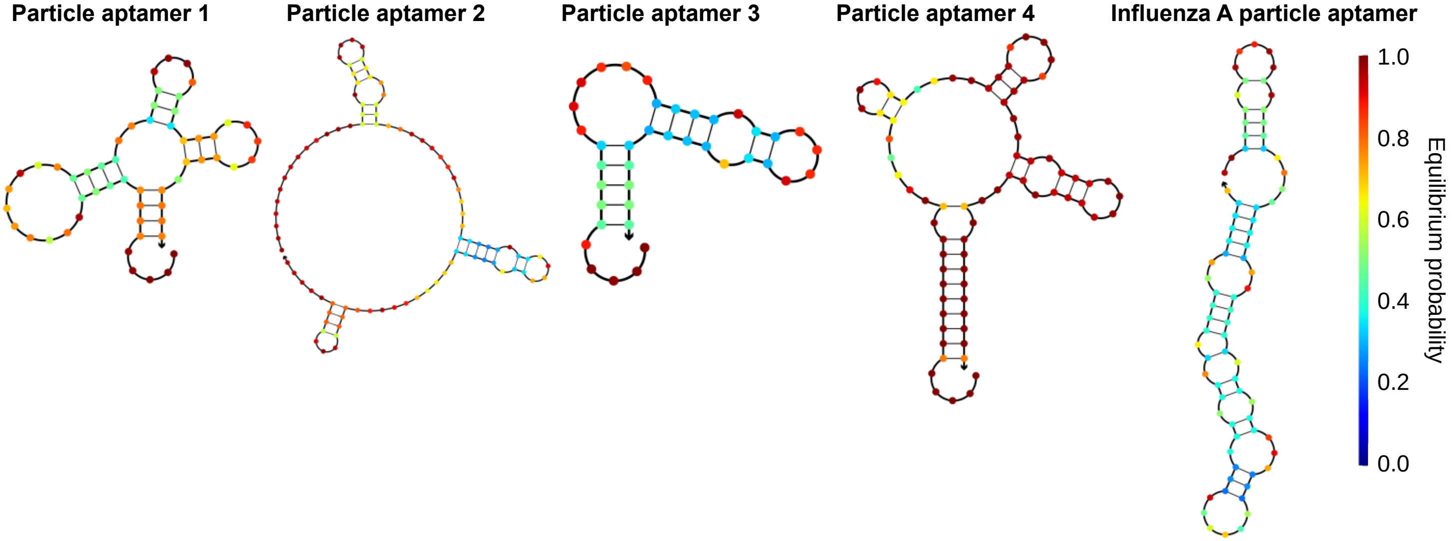

Figure S1. Secondary structure prediction and alignment of aptamers. (**a**) Secondary structures were predicted using NUPACK (http://www.nupack.org/) for aptamers 1, 2, 3, 4 and influenza A aptamer with the following conditions: 25˚C, 0.137 M Na^{+}. Arrows represent the 3' end of the aptamer. Nucleotides are color-coded by their equilibrium probability (legend on right).

Figure S2. Fluorescence imaging of Rolosense chip with aptamer.

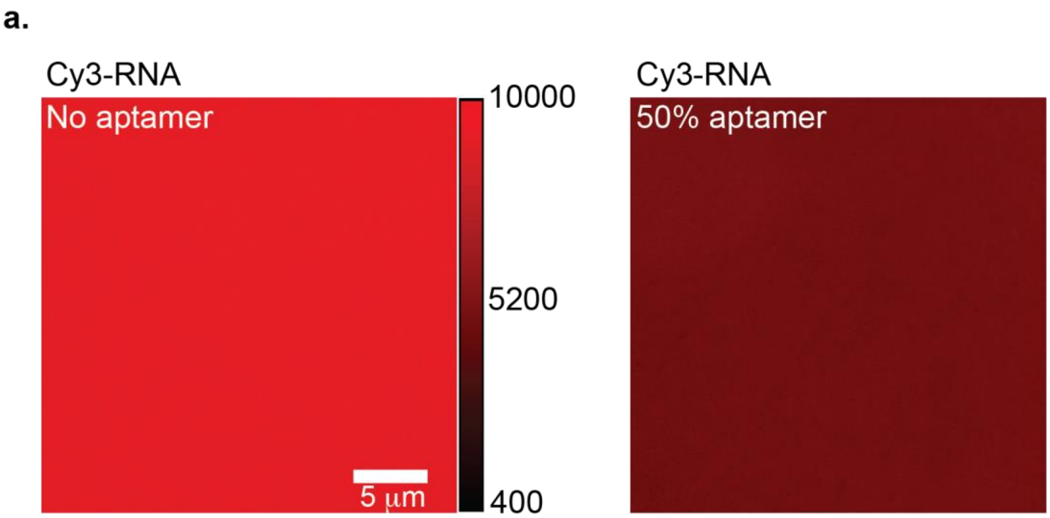

Figure S2. Fluorescence imaging of Rolosense chip with aptamer. **a,** Cy3 fluorescence images of chip surfaces with no aptamer and 50% aptamer 1. The vertical color bar indicates the minimum, median, and maximum values (a.u.) used to indicate the fluorescence of the Cy3-tagged RNA. The fluorescence intensity of the Rolosense chip with 50% aptamer 1 is reduced by half compared to the chip with no aptamer (100% Cy3-tagged RNA).

Figure S3. DNA-based motors tolerate certain densities of aptamer.

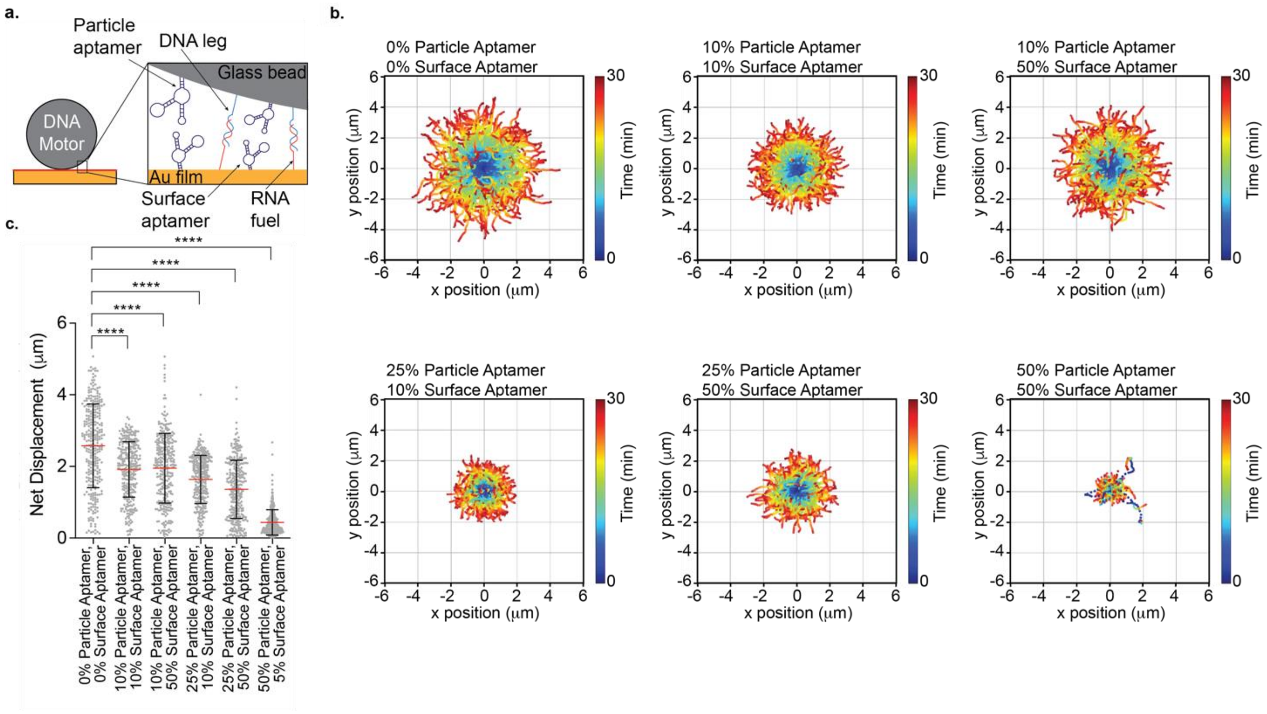

Figure S3. DNA-based motors tolerate certain densities of aptamer. a, Schematic of DNA motor and chip comprised of particle aptamer, DNA leg, surface aptamer, RNA fuel respectively. DNA motors are modified with a binary mixture of DNA leg and particle aptamer. The Rolosense chip is modified with a binary mixture of RNA fuel and surface aptamer. **b,** Plots showing the trajectory of motors with different densities of particle and surface aptamer 1. All the trajectories are aligned to the 0,0 (center) of the plots for time = 0 min. Color indicates time (0 → 30 min). **c,** Plots of net displacement of ~300 motors with 0% particle aptamer, 0% surface aptamer; 10% particle aptamer, 10% surface aptamer; 10% particle aptamer, 50% surface aptamer; 25% particle aptamer, 10% surface aptamer; 25% particle aptamer, 50% surface aptamer; 50% particle aptamer, 5% surface aptamer. 0% particle aptamer and 0% surface aptamer serves as the control with only DNA leg and RNA fuel. **** indicates $P < 0.0001$.

Figure S4. Quantification of VLPs using single particle imaging.

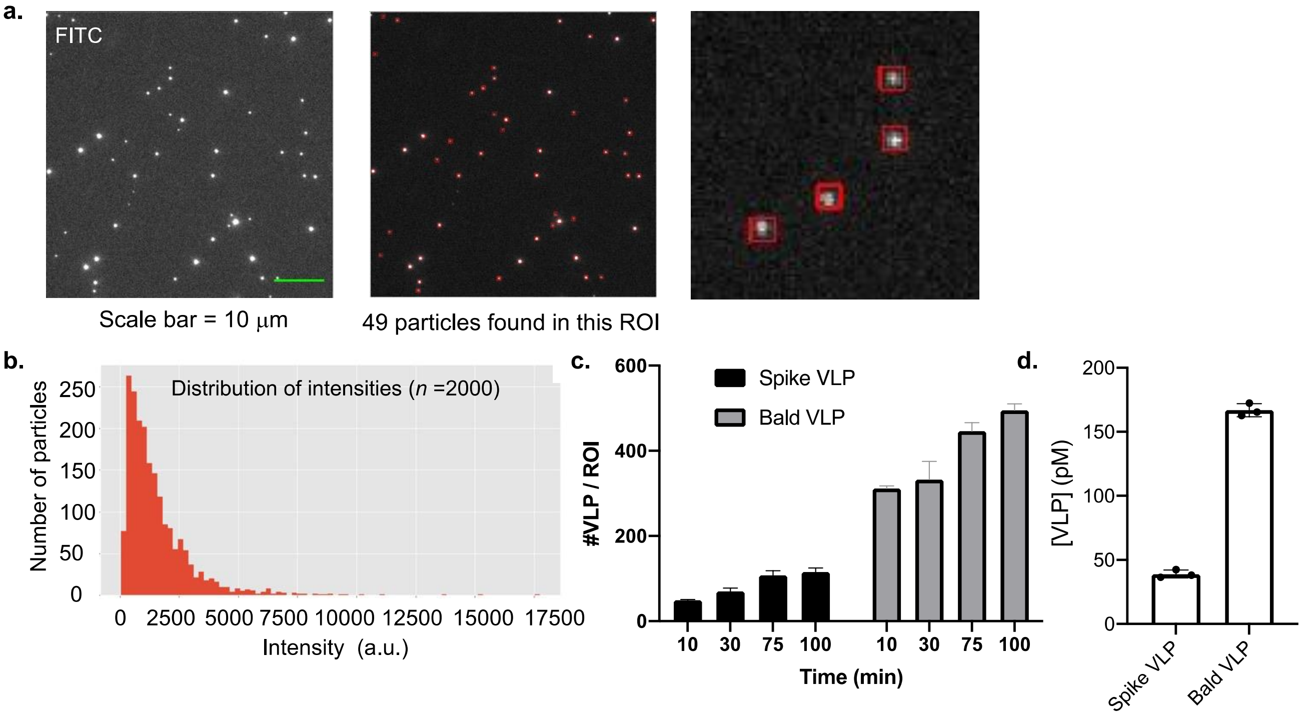

Figure S4. Quantification of VLPs using single particle imaging. a, Fluorescence imaging of VLPs on an amine modified glass slide. **b,** Distribution of the fluorescence intensity of 2,000 particles. **c,** Quantification of VLPs per ROI to determine density of particles per μm^2. **d,** Determined concentration of stock VLP samples which was obtained using the area of the imaging channel (6.46×10^7 μm^2) and total volume of VLP samples (100 μL): $\frac{\#\, of\, VLPs\, per\, ROI * 6.46x10^7\, \mathrm{um}^2}{100\, \mathrm{uL}}$.

Figure S5. Net displacement of aptamer-modified motors without VLPs.

a.

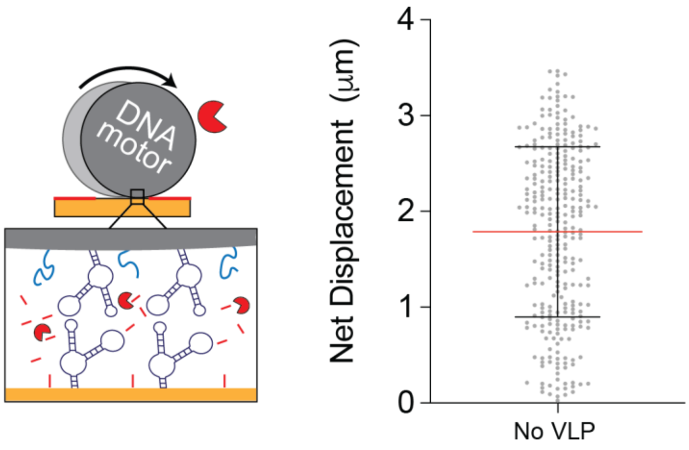

Figure S5. Net displacement of aptamer-modified motors without VLPs. a, (Left) Schematic of DNA motor and chip functionalized with aptamer 1. (Right) Plot showing net displacement of over 100 motors with no VLP. Experiments were done in triplicate and the net displacement for these motors was 1.87 μm +/- 0.88 μm.

Figure S6. Using motors modified with antibodies to detect SARS-CoV-2.

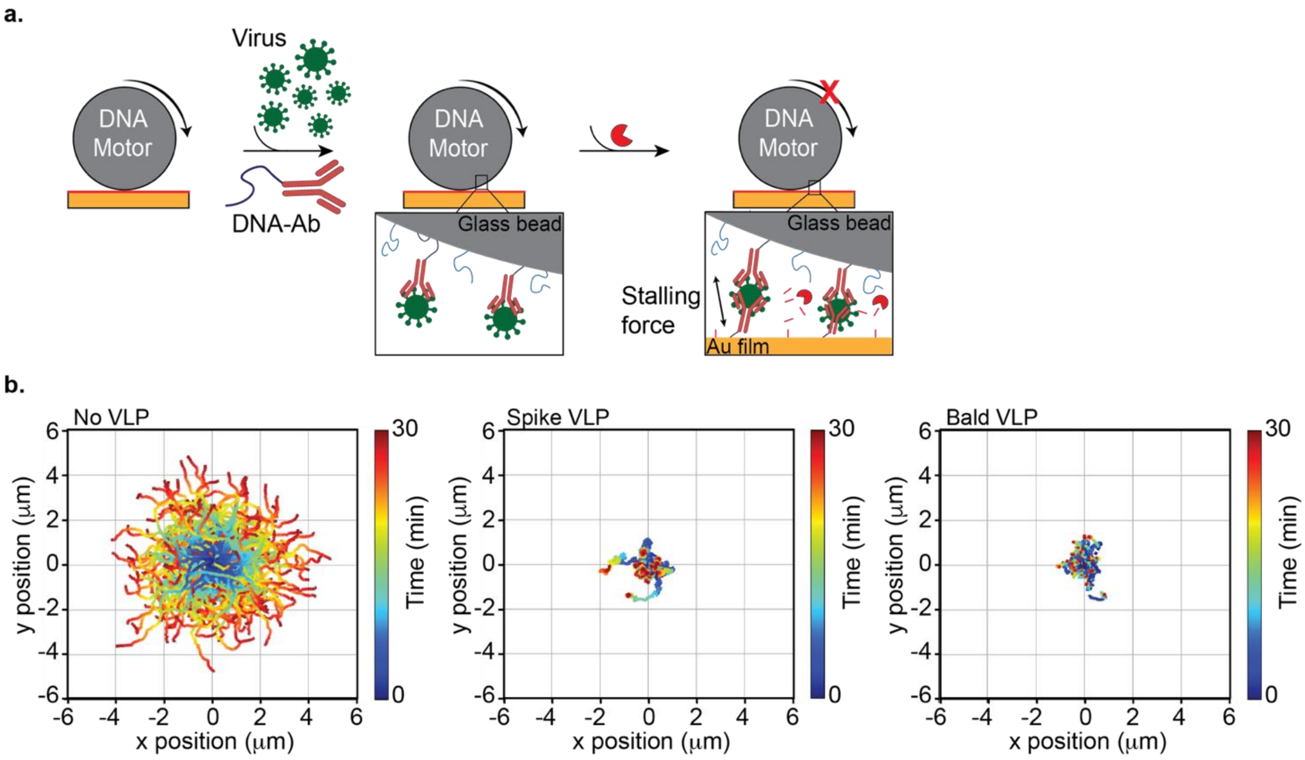

Figure S6. Using motors modified with antibodies to detect SARS-CoV-2. a, Schematic of Rolosense detection of SARS-CoV-2 virus particles using a commercially available antibody with high affinity for S1 subunit of spike protein. The DNA-S1 antibody conjugate was added to the motors and chip via hybridization. **b,** Plots showing the trajectory of motors without VLP, 25pM spike VLP, and 25pM bald VLP. All the trajectories are aligned to the 0,0 (center) of the plots for time = 0 min. Color indicates time (0 → 30 min). The DNA motors modified with antibodies did not show a specific response to the spike protein detection.

Figure S7. DNA-based motors tolerate artificial saliva as a medium for virus sensing.

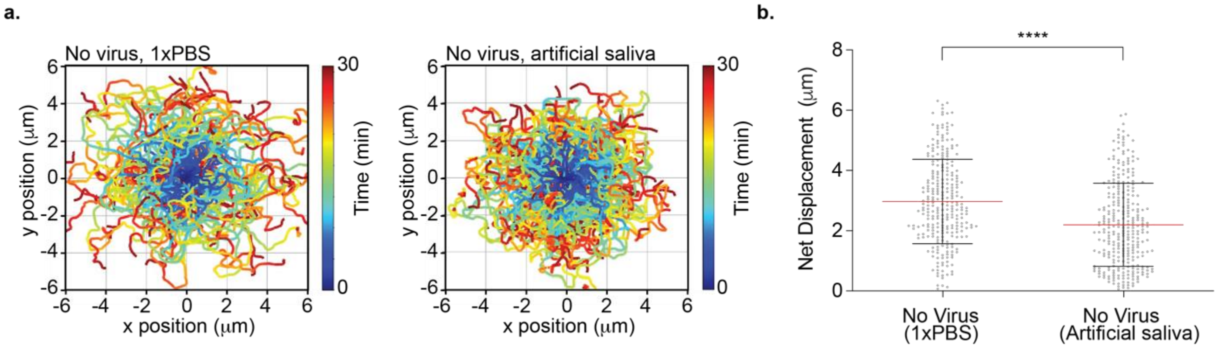

Figure S7. DNA-based motors tolerate artificial saliva as a medium for virus sensing. a, Plots showing the trajectory of motors without virus diluted in 1xPBS (phosphate-buffered saline) and artificial saliva. All the trajectories are aligned to the 0,0 (center) of the plot for time = 0 min. Color indicates time (0 → 30 min). The motors were modified with 10% aptamer 3 and the chip with 50% aptamer 3. **b,** Plots of net displacement of ~300 motors without virus diluted in 1xPBS and artificial saliva. **** indicates $P < 0.0001$. Experiments were performed in triplicate.

Figure S8. Detecting SARS-CoV-2 WA-1 virus in artificial saliva.

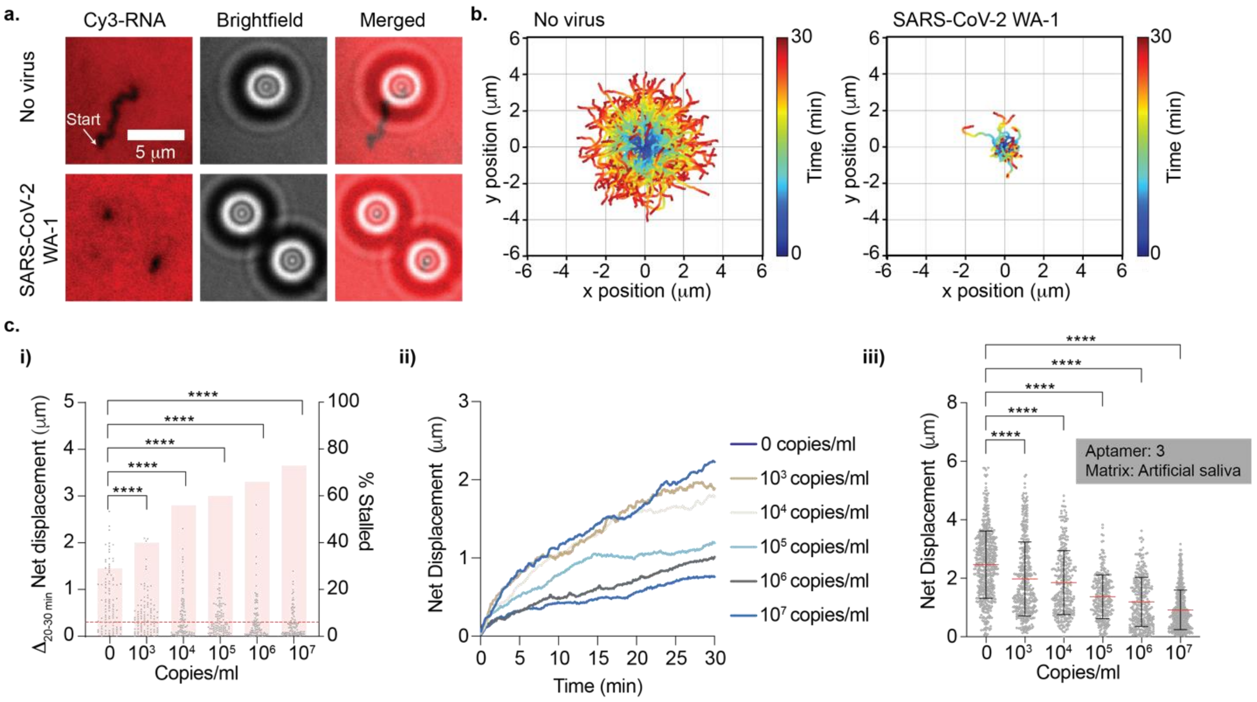

Figure S8. Detecting WA-1 SARS-CoV-2 virus in artificial saliva. a, Fluorescence and brightfield imaging of DNA motors detecting the presence of 10^8 copies/mL of UV-inactivated SARS-CoV-2 WA-1 spiked in artificial saliva. DNA motors were incubated for 30 min with the virus samples. Samples with SARS-CoV-2 show stalled motors and no depletion tracks in contrast to samples lacking the virus. **b,** Plots showing the trajectories of motors with no virus and 10^8 copies/mL of UV-inactivated SARS-CoV-2 WA-1 strain spiked in artificial saliva. All the trajectories are aligned to the 0,0 (center) of the plots for time = 0 min. Color indicates time (0 → 30 min). **c, i)** Plots of the Δnet displacement as well as the percentage of motors stalled in the last 10 minutes of the 30-minute time lapse for 100 motors incubated with ranging concentrations of SARS-CoV-2 WA-1. To calculate a percentage of stalled motors, 0.300 µm threshold was used (represented by red dotted line). **ii)** Plots of the net displacement over the 30-minute time lapse for 30 motors incubated with different concentrations of SARS-CoV-2 WA-1. **iii)** Plots of net displacement of over 300 motors for 3 independent replicates. The error bars and the red lines represent the standard deviation and the mean of the distribution, respectively. UV-inactivated SARS-CoV-2 samples were spiked in artificial saliva and incubated with the motors functionalized with aptamer 3 at room temperature for 30 min. **** indicates $P < 0.0001$.

Figure S9. DNA-based motors incubated with virus bind specifically to aptamers on the Rolosense chip.

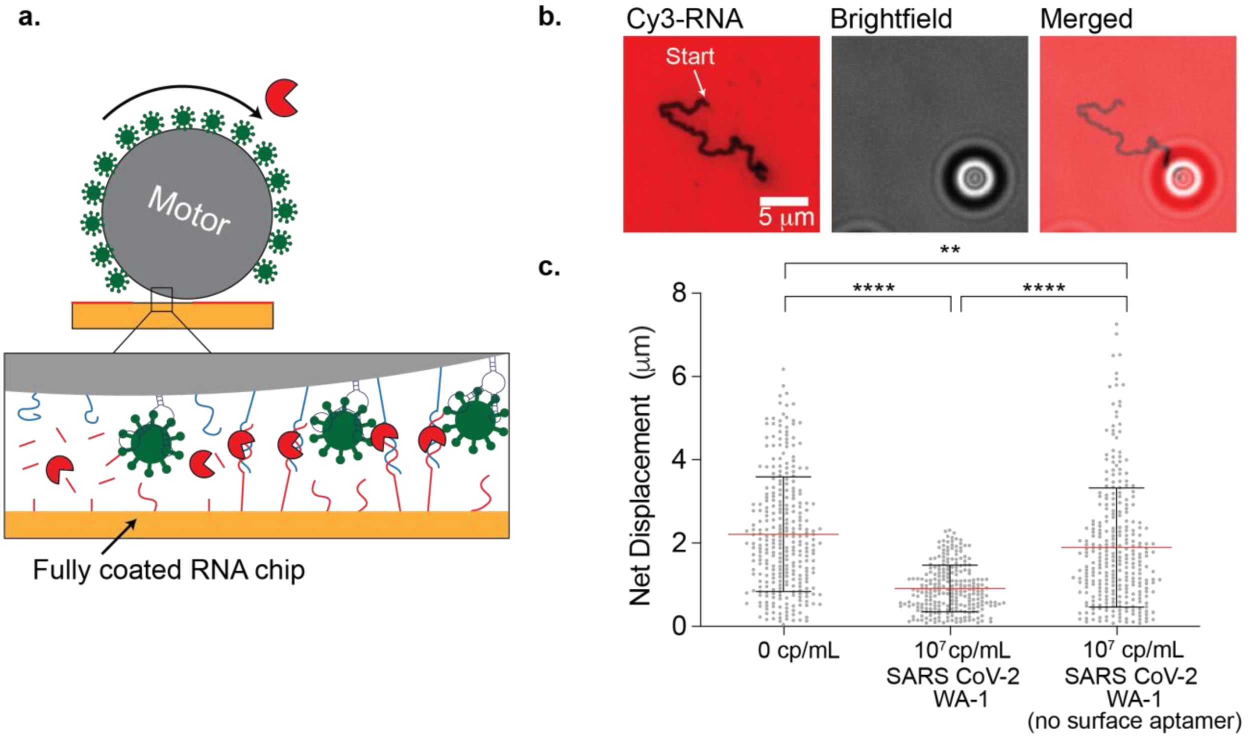

Figure S9. DNA-based motors incubated with virus bind specifically to aptamers on the Rolosense chip. a, Schematic of DNA motor functionalized with aptamer 3 rolling on a fully coated RNA chip (no aptamer) in the presence of the RNaseH. **b,** Fluorescence and brightfield imaging of DNA motors modified with 10% aptamer 3 incubated with 10^7 copies/mL of UV-inactivated SARS-CoV-2 WA-1 spiked in 1xPBS for 30 mins at room temperature. The motors formed long RNA depletion tracks on the chip that was not functionalized with surface aptamer. The RNA fuel was tagged with Cy3, shown here in red. **c,** Plots of net displacement of ~300 motors with 10% particle aptamer, 50% surface aptamer with no virus; 10% particle aptamer, 50% surface aptamer with 10^7 copies/mL of UV-inactivated SARS-CoV-2 WA-1; and 10% particle aptamer, 0% surface aptamer with 10^7 copies/mL of UV-inactivated SARS-CoV-2 WA-1. ** and **** indicate $P<0.01$ and $P<0.0001$, respectively. Experiments were performed in triplicate.

Figure S10. Eliminating washing steps does not affect the integrity of Rolosense.

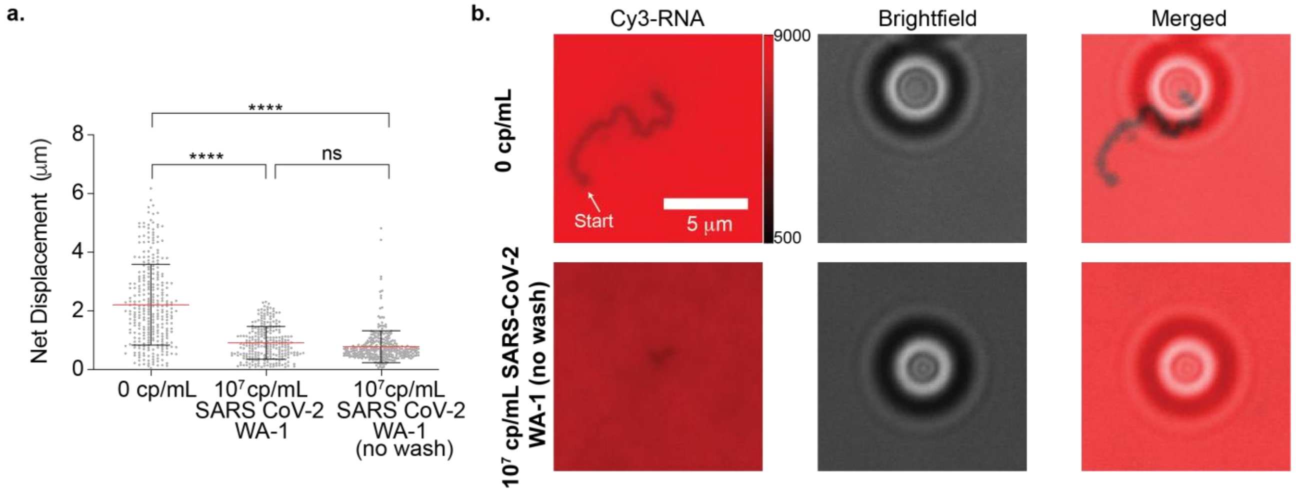

Figure S10. Eliminating washing steps does not affect the integrity of Rolosense. a, Plots of net displacement of ~300 motors with no virus, 10^7 copies/mL of UV-inactivated SARS-CoV-2 WA-1 in artificial saliva, and 10^7 copies/mL of UV-inactivated SARS-CoV-2 WA-1 in artificial saliva with no washing step following virus incubation with the DNA motors. **** and ns indicate $P < 0.0001$ and not statistically significant, respectively. Experiments were performed in triplicate. **b,** Fluorescence and brightfield imaging of DNA motors without virus diluted in artificial saliva. The motors form depletion tracks in the Cy3-RNA channel in the presence of RNaseH. When the DNA motors are incubated with 10^7 copies/mL of SARS-CoV-2 WA-1 spiked in artificial saliva without the washing step, they do not form depletion tracks in the Cy3-RNA channel. However, foregoing the wash after sample incubation does not compromise the integrity of RNA on the chip and Rolosense continues to function. The vertical color bar indicates the minimum and maximum values (a.u.) used to display the fluorescence of the Cy3-tagged RNA. We include this bar to note the integrity of the RNA monolayer.

Figure S11. Decreasing DNA motor and virus incubation time does not affect the overall performance of Rolosense.

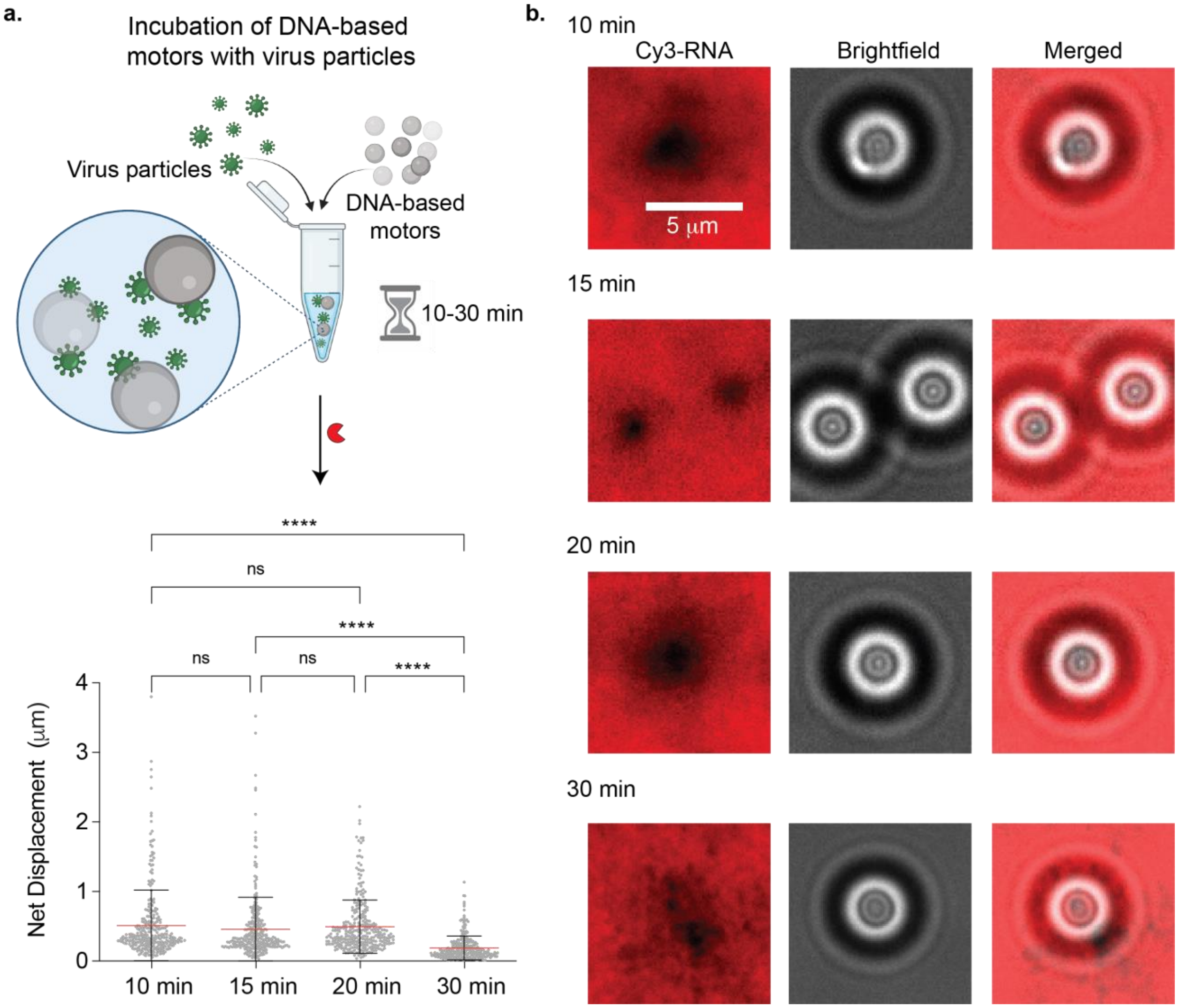

Figure S11. Decreasing DNA motor and virus incubation time does not affect the overall performance of Rolosense. a, (Top) Schematic of sample generation which includes incubation of virus with DNA motors ranging from 10 to 30 mins. (Bottom) Plots of net displacement of ~300 motors with 10^7 copies/mL of UV-inactivated SARS-CoV-2 BA-1 in 1xPBS at different sample incubation time points following RNAseH addition. ns and **** indicate not statistically significant and $P < 0.0001$, respectively. Experiments were performed in triplicate. **b,** Fluorescence and brightfield imaging of DNA motors incubated with 10^7 copies/mL of SARS-CoV-2 BA.1 at different sample incubation time points. No depletion tracks are observed for samples incubated at 10 min, 15 min, 20 min, or 30 min.

Figure S12. Motor trajectories over time as a function of increasing virus concentration.

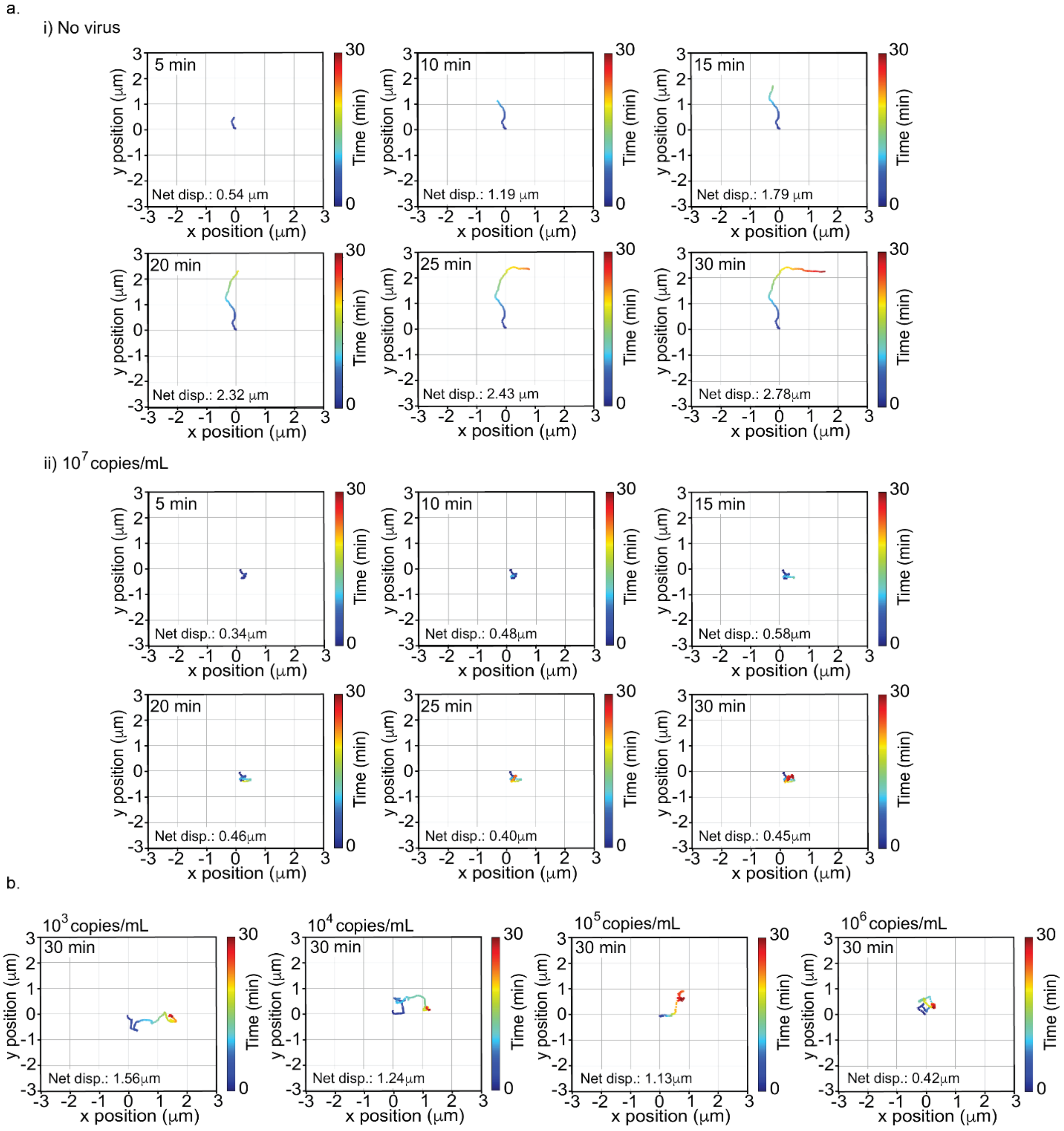

Figure S12. Motor trajectories over time as a function of increasing virus concentration. a, Representative trajectory plots of single motors with no virus and motors incubated with 10^7 copies/mL of SARS-CoV-2 B.1.617.2 at 5, 10,15, 20, 25, and 30 mins. All the trajectories are aligned to the 0,0 (center) of the plots for time = 0 min. Color indicates time (0 → 30 min). If the motor with virus never encounters an aptamer on the surface, its trajectory grows, and the net displacement increases over time. However, the encounter and binding to one or more aptamers on the surface lead to motor

stalling, as indicated by the abrupt red motor coordinates at the tip of the trajectories. **b,** Representative trajectory plots of single motors incubated with 10^3,10^4,10^5, and 10^6 copies/mL of SARS-CoV-2 B.1.617.2 at 30 mins. All the trajectories are aligned to the 0,0 (center) of the plots for time = 0 min. Color indicates time (0 → 30 min).

Figure S13. Modeling motor-virus encounter rates.

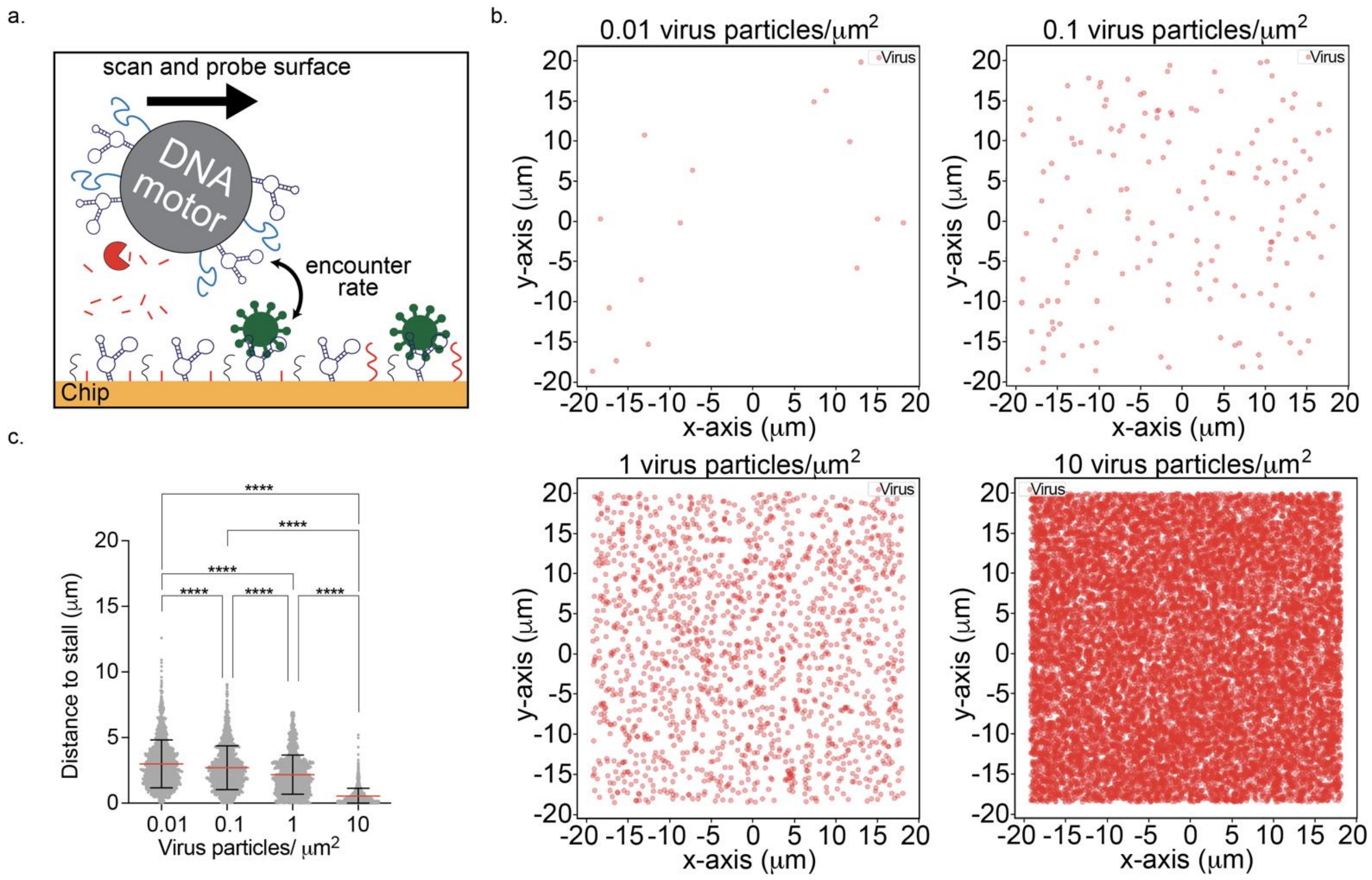

Figure S13. Modeling motor-virus encounter rates. a, Schematic of the modeling setup in which DNA motors scan and probe the surface for virus particles that are bound to aptamer on the chip. Upon encountering viruses on the chip, the DNA motors stall. **b,** Virus immobilization on a 40x40 micron chip surface at different concentrations. Virus positions were generated at random. Each red dot is a virus particle. **c,** Plots of the distance that it takes the motors (*N*= 1200) to stall upon virus encounter at different virus concentrations in the 30-min period. The model is based on real motor trajectory data taken from the 'no virus' control to simulate motor motion in the presence of 0.01, 0.1, 1, and 10 virus particles/ μm^2. A motor is considered stalled when it comes within 90 nm of a virus particle, with a 100% stalling probability under these conditions. The motors show a distribution in motion with some motors never meeting a virus and continuing to move normally, while others do encounter a virus and stall. The error bars and the red lines represent the standard deviation and the mean of the distribution, respectively. **** indicates $P < 0.0001$.

Figure S14. Detection sensitivity of SARS-CoV-2 in artificial saliva.

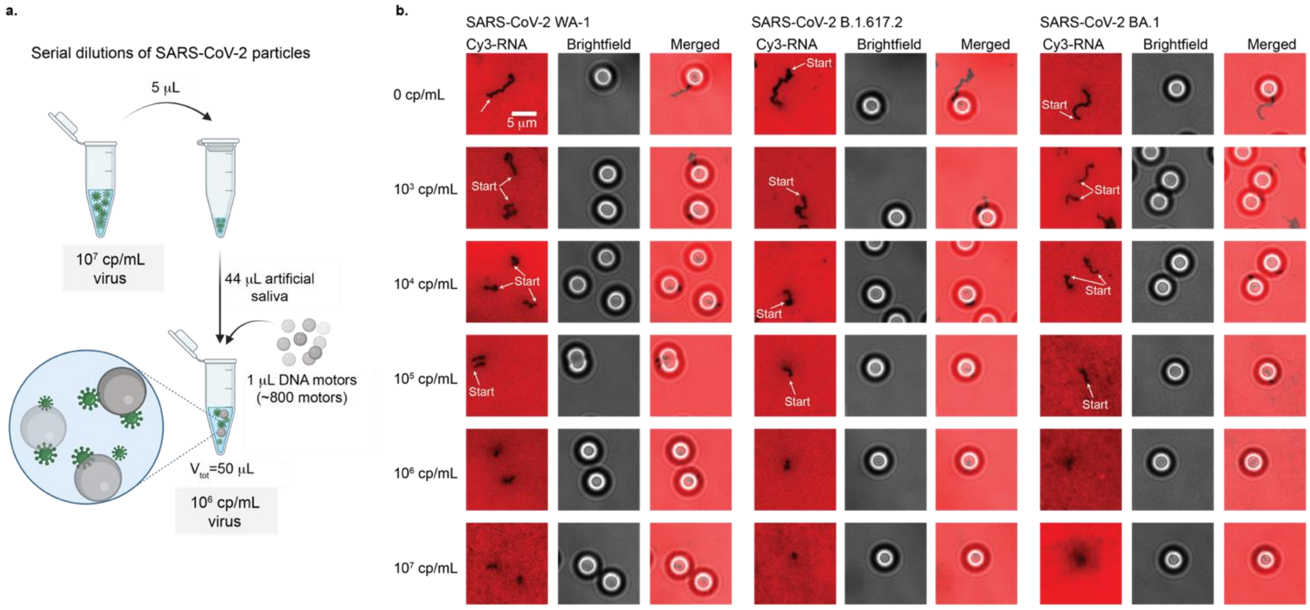

Figure S14. Detection sensitivity of SARS-CoV-2 in artificial saliva. a, Schematic of artificial saliva sample generation. Serial dilutions of SARS-CoV-2 particles are created in a 50 μL total volume which includes 44 μL of artificial saliva and 1 μL of DNA motors (~800 motors). **b,** Fluorescence and brightfield imaging of DNA motors incubated with different concentrations of UV-inactivated SARS-CoV-2 WA-1, B.1.617.2, and BA.1 spiked in artificial saliva. For all three variants, the DNA motors display decreasing depletion track length in the Cy3-RNA channel as virus concentration increases.

Figure S15. Detecting SARS-CoV-2 BA.1 strain in artificial saliva.

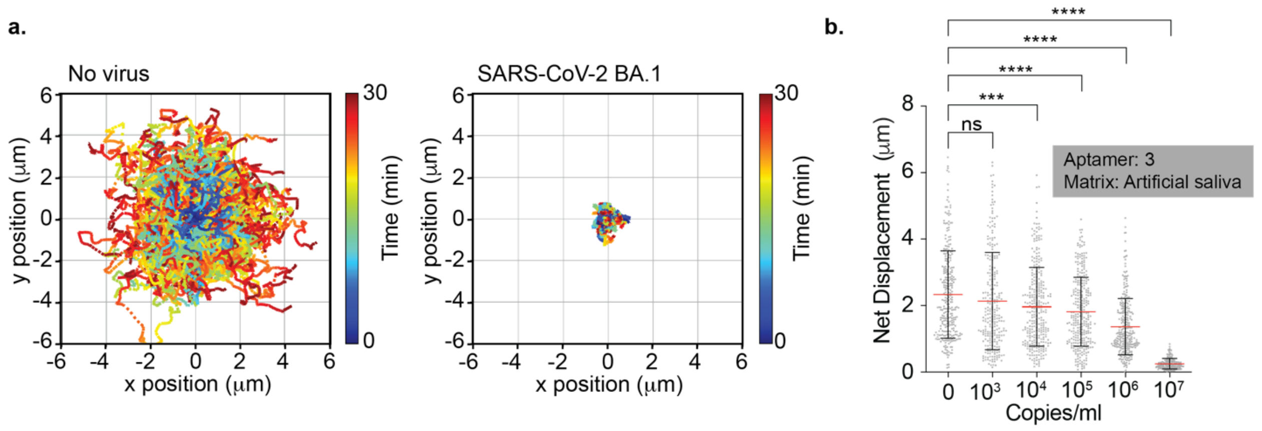

Figure S15. Detecting SARS-CoV-2 BA.1 strain in artificial saliva. a, Plots showing the trajectories of motors with no virus and 10^7 copies/mL of UV-inactivated SARS-CoV-2 BA.1 strain spiked in artificial saliva. All the trajectories are aligned to the 0,0 (center) of the plots for time = 0 min. Color indicates time (0 → 30 min). **b**, Plots of net displacement of over 300 motors for 3 independent replicates. The error bars and the red lines represent the standard deviation and the mean of the distribution, respectively. UV-inactivated SARS-CoV-2 samples were spiked in artificial saliva and incubated with the motors functionalized with aptamer 3 at room temperature for 30 min. ns, ***, **** indicate not statistically significant, $P < 0.001$, and $P < 0.0001$.

Figure S16. Motors demonstrate a specific response to SARS-CoV-2 viruses.

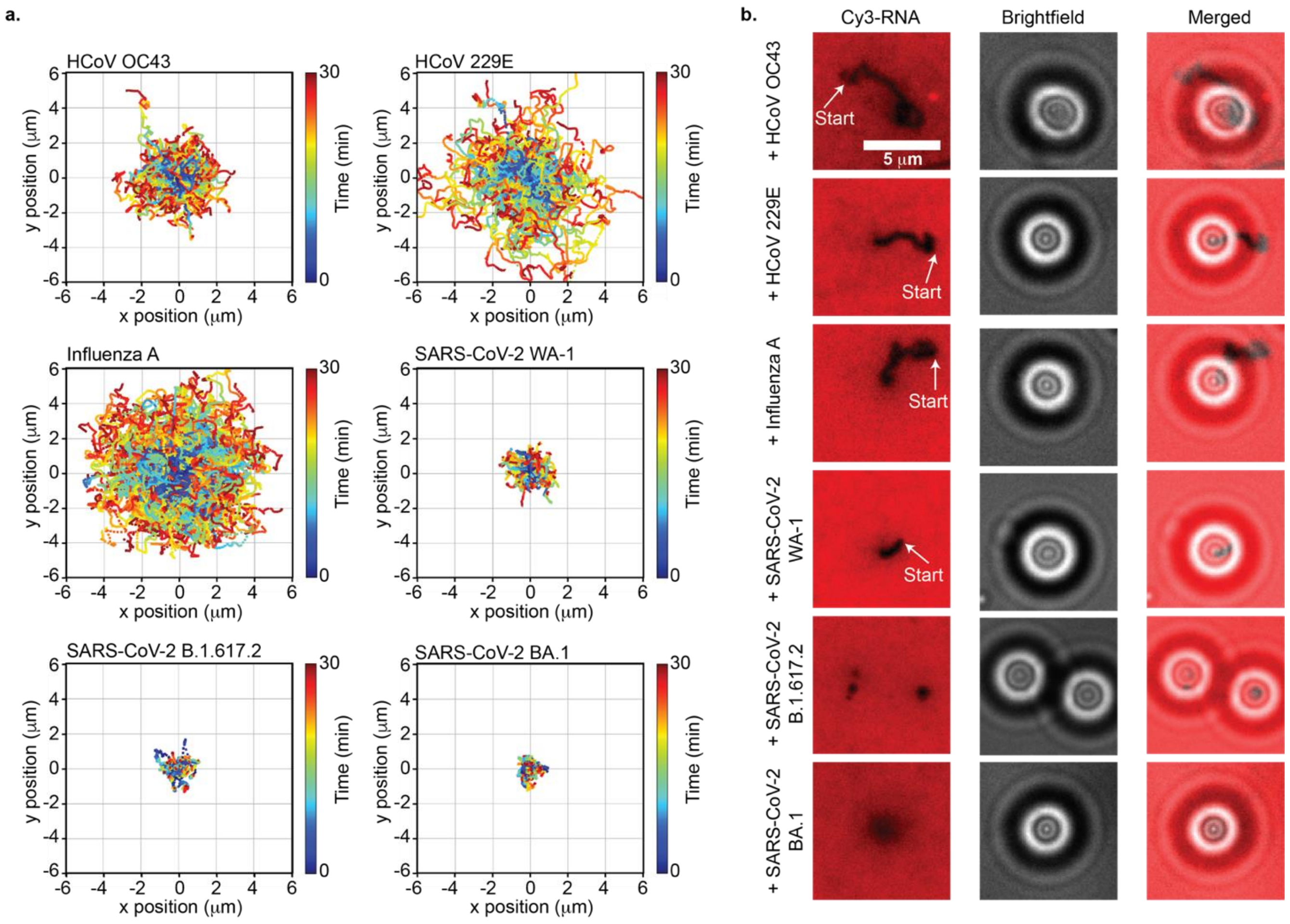

Figure S16. Motors demonstrate a specific response to SARS-CoV-2 viruses. a, Plots showing the trajectory of motors incubated with 10^7 copies/mL of UV-inactivated HCoV OC43, HCoV 229E, influenza A, SARS-CoV-2 WA-1, B.1.617.2, and BA.1 in artificial saliva. All the trajectories are aligned to the 0,0 (center) of the plots for time = 0 min. Color indicates time (0 → 30 min). **b,** Fluorescence and brightfield imaging of DNA motors with 10^7 copies/mL of UV-inactivated HCoV OC43, HCoV 229E, influenza A, SARS-CoV-2 Washington WA-1, SARS-CoV-2 B.1.617.2, and SARS-CoV-2 BA.1 spiked in artificial saliva. Motors incubated with UV-inactivated SARS-CoV-2 WA-1, B.1.617.2, and BA.1 showed no RNA depletion tracks whereas motors incubated with UV-inactivated HCoV OC43, HCoV 229E, and influenza A formed long RNA depletion tracks.

Figure S17. DNA motors detect influenza A virus.

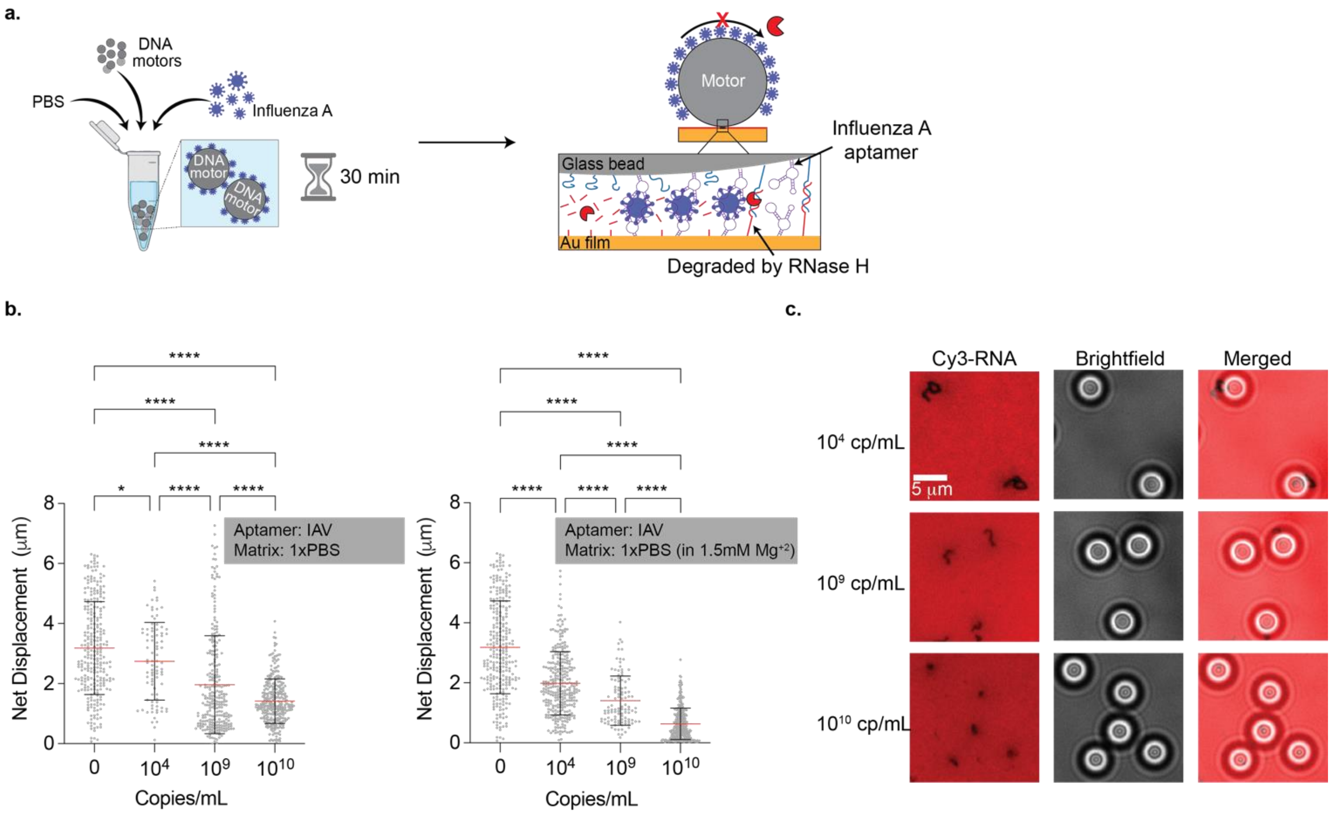

Figure S17. DNA motors detect influenza A virus. a, Schematic of sample generation for influenza A detection. DNA motors functionalized with 10% influenza A aptamer are incubated with influenza A virus in 1xPBS for 30 mins at room temperature. When added to the RNA chip decorated with 50% of the same aptamer, the motors stalled in the presence of RNAseH. **b,** Plot on the left shows the net displacement of >100 motors incubated with different concentrations of influenza A virus in 1xPBS. Plot on the right shows the net displacement of >100 motors incubated with different concentrations of influenza A virus in 1xPBS supplemented with 1.5mM Mg^{+2}. * and **** indicate $P < 0.05$ and $P < 0.0001$, respectively. Experiments were performed in triplicate. **c,** Fluorescence and brightfield imaging of DNA motors with different concentrations of influenza A virus spiked in 1xPBS supplemented with 1.5 Mg^{+2}. RNA depletion tracks formed by the motors decrease as influenza A virus concentration increases.

Figure S18. DNA motors tolerate breath condensate as a virus sensing medium.

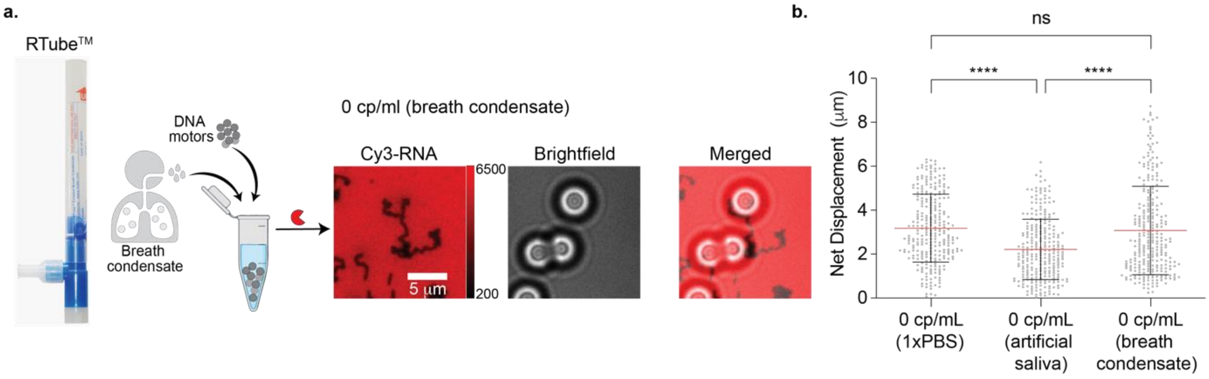

Figure S18. DNA motors tolerate breath condensate as a virus sensing medium. a, Schematic of sample generation in exhaled breath condensate using the RTube™. After collection, 1 μL of motors were mixed in the breath condensate sample. Fluorescence and brightfield imaging show that DNA motors diluted in breath condensate without virus display long depletion tracks on Rolosense chip with aptamer. The vertical color bar indicates the minimum and maximum values (a.u.) used to display the fluorescence of the Cy3-tagged RNA. We include this bar to note the integrity of the RNA monolayer. **b,** Plots of net displacement of ~300 motors with no virus diluted in 1xPBS, artificial saliva, and breath condensate. ns and **** indicate not statistically significant and $P < 0.0001$, respectively. Experiments were performed in triplicate.

Figure S19. Blinded limit of detection (LoD) challenge panel.

a.

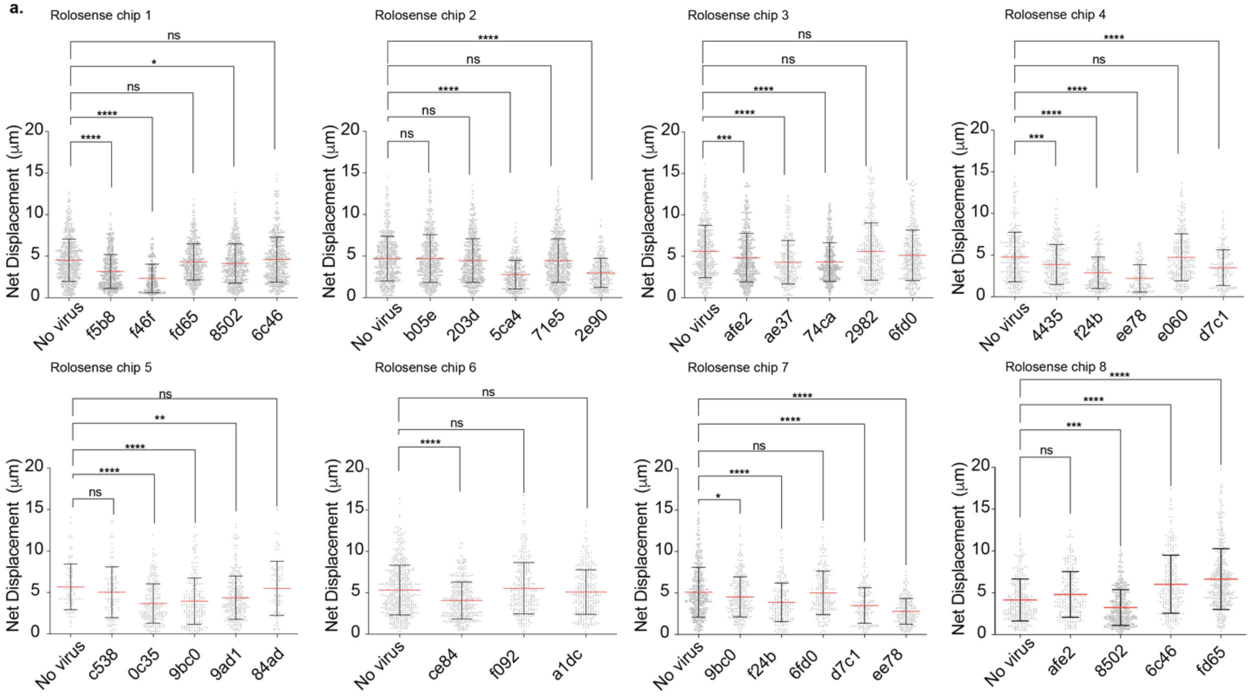

b.

Sample ID	% displacement from control ((Net disp.$_{sample}$/ Net disp.$_{no\ virus}$)x100)	Concentration (cp/mL) *blinded*	+/- *blinded*	Concentration (cp/mL) *unblinded*	+/- *unblinded*
f46f	51.620	$1.02x10^{5}$	+	$6.40x10^{3}$	+
5ca4	58.832	$1.02x10^{5}$	+	$4.00x10^{2}$	-
2e90	63.248	$1.02x10^{5}$	+	$6.40x10^{3}$	+
4435	64.774	$5.12x10^{4}$	+	$5.12x10^{4}$	+
d7c1	68.615	$5.12x10^{4}$	+	$1.02x10^{5}$	+
ee78	69.542	$5.12x10^{4}$	+	0	-
f5b8	70.293	$2.56x10^{4}$	+	$2.56x10^{4}$	+
f24b	75.912	$2.56x10^{4}$	+	$5.12x10^{4}$	+
ce84	76.625	$2.56x10^{4}$	+	$1.60x10^{3}$	+
ae37	76.650	$6.40x10^{3}$	+	$1.60x10^{3}$	+
6fd0	76.957	$6.40x10^{3}$	+	$2.56x10^{4}$	+
74ca	77.169	$6.40x10^{3}$	+	$1.02x10^{5}$	+
8502	78.397	$1.60x10^{3}$	+	$5.12x10^{4}$	+
c538	88.646	$1.60x10^{3}$	+	$1.60x10^{3}$	+
9bc0	88.956	$1.60x10^{3}$	+	$6.40x10^{3}$	+
9ad1	91.697	$4.00x10^{2}$	-	0	-
203d	94.543	$4.00x10^{2}$	-	$2.50x10^{1}$	-
71e5	94.692	$4.00x10^{2}$	-	$4.00x10^{2}$	-
a1dc	95.640	$1.00x10^{2}$	-	$4.00x10^{2}$	-
fd65	95.870	$1.00x10^{2}$	-	$2.50x10^{1}$	-
84ad	96.773	$1.00x10^{2}$	-	$2.56x10^{4}$	+
b05e	99.830	$2.50x10^{1}$	-	$1.00x10^{2}$	-
2982	99.910	$2.50x10^{1}$	-	$1.02x10^{5}$	+
6c46	101.931	$2.50x10^{1}$	-	$1.00x10^{2}$	-
f092	104.265	0	-	$2.50x10^{1}$	-
afe2	115.906	0	-	0	-
0c35	121.737	0	-	$1.00x10^{2}$	-
e060	148.400	0	-	0	-

Figure S19. Blinded limit of detection (LoD) challenge panel. a, Plots of net displacements of ~300 motors diluted in blinded SARS-CoV-2 BA.1 samples. The blinded SARS-CoV-2 BA.1 samples were prepared in breath condensate. The DNA-based motors and Rolosense chips were modified with aptamer 4. After 30 mins, the motors are added to Rolosense chip and imaged for 30 mins. Each Rolosense chip tested included a ‘no virus’ control in which the motors were diluted in breath condensate without any virus. Samples with lower particle count than the no virus control were repeated. The error bars represent the standard deviation, and the red lines represent the mean of the distribution. ns, *, **, *** and **** indicate not statistically significant, $P < 0.05$, $P < 0.01$, $P < 0.001$, and $P < 0.0001$, respectively. **b,** Table of % displacement of control, blinded concentration values, blinded +/- call, unblinded concentration values, and unblinded +/- call for each sample tested. We normalized each sample by the ‘no virus’ control for that Rolosense chip and we termed this ‘% displacement from control.’ The samples were then sorted by increasing % displacement values and assigned concentration values. The smaller the % displacement value, the more virus the sample has. Each sample was also assigned positive (+) and negative (-) calls based on 1.00×10^3 copies/mL limit of detection (LoD). The true concentration values were then unblinded and assigned positive (+) and negative (-) calls using the same LoD threshold based on these values. Based on the true positive (+) and negative (-) calls from the unblinded concentrations, we identified 2 false positives and 2 false negatives (highlighted in red).

Figure S20. DNA-based motors stall in the presence of a single viral particle.

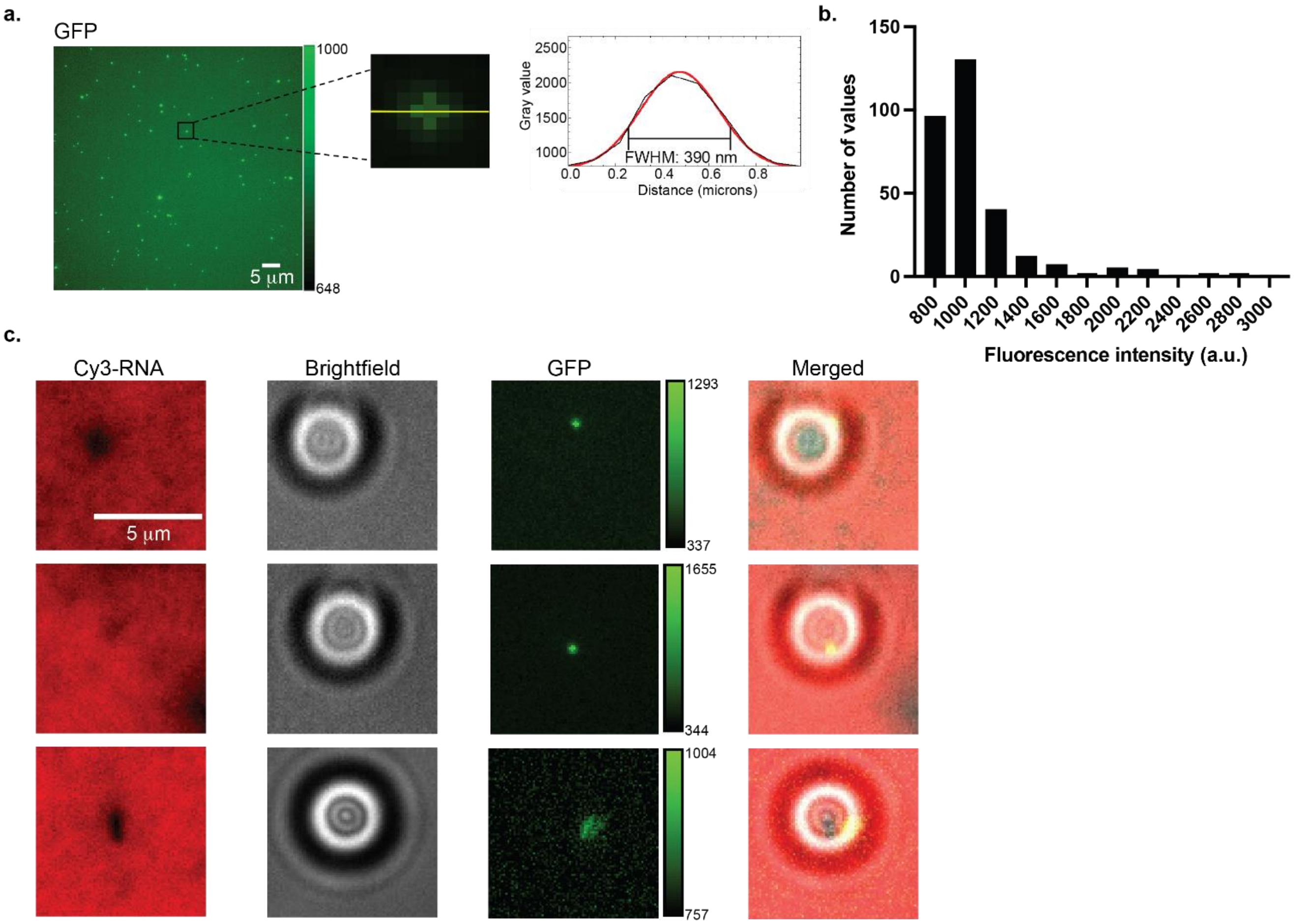

Figure S20. DNA-based motors stall in the presence of a single viral particle. a, Fluorescence imaging of 1000x diluted spike VLP particles (48 fM in 1xPBS) on the Rolosense chip. Line scan drawn on one of the particles indicate that the full width half maximum (FWHM) of a single particle is 390 nm. **b,** Distribution of the fluorescence intensity of 1000x diluted spike VLP particles in 1xPBS on Rolosense chip. Single VLP particles have a fluorescence intensity of 800-1200 a.u. **c,** Brightfield and fluorescence imaging of DNA motors incubated with 25 pM of GFP-labeled spike VLPs in 1xPBS. Samples with GFP-labeled spike VLPs show stalled motors and no depletion tracks along with a colocalized GFP signal with fluorescence intensity values indicating single particle. The vertical color bars indicate the minimum and maximum values (a.u.) used to display the fluorescence of the GFP-tagged VLPs. We include these bars to note the quantitative variations in fluorescence intensity of the bound VLPs.

Figure S21. Rolosense chip can tolerate saliva in the presence of RNase inhibitors.

a.

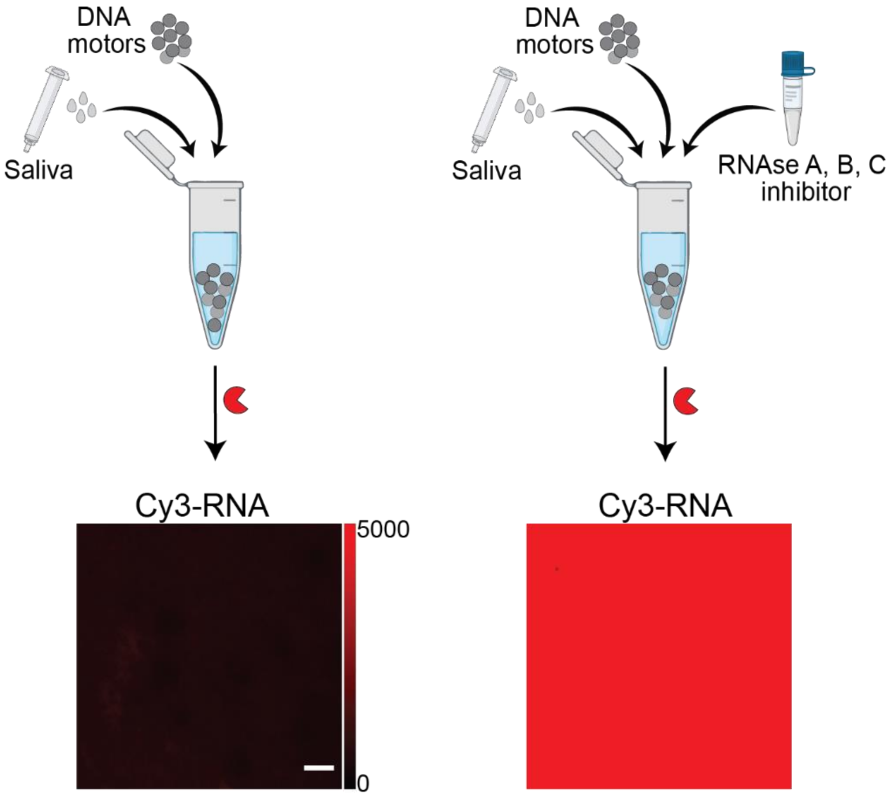

Figure S21. Rolosense chip can tolerate saliva in the presence of RNase inhibitors. a, DNA motors were incubated with saliva for 30 mins at room temperature and washed 3x in 1xPBS via centrifugation (15,000 rpm, 1 min). After wash the motors were added to Rolosense chip in the presence of RNaseH enzyme. Following 30 mins of RNaseH enzyme incubation, the Cy3 fluorescence intensity dropped significantly due to nuclease degradation. The Cy3 fluorescence intensity was recovered by incubating the motors for 30 mins at room temperature in saliva along with 20 units of RNase A, B, and C inhibitor. After the incubation, the sample was washed 3x in 1xPBS via centrifugation (15,000 rpm, 1 min) and added to the Rolosense chip along with RNaseH. The vertical color bar indicates the minimum and maximum values (a.u.) used to display the fluorescence of the Cy3-tagged RNA. We include this bar to note the integrity of the RNA monolayer following exposure to saliva. Scale bar is 10 μm.

Supplementary Movies

Supplementary Movie S1: Timelapse video of the brightfield channel superimposed with color-coded particle trajectories acquired at 5 s intervals for a duration of 30 mins. The video was acquired following RNaseH addition using a 20x 0.5 NA objective. SARS-CoV-2 and influenza A motors are not incubated with virus and both motors displayed long trajectories. The SARS-CoV-2 motors (6μm polystyrene) are distinguishable from the influenza A motors (5μm silica) on brightfield due to their darker outer rim. Scale bar is 10 μm.

Supplementary Movie S2: Timelapse video of the brightfield channel superimposed with color-coded particle trajectories acquired at 5 s intervals for a duration of 30 mins. The video was acquired following RNaseH addition using a 20x 0.5 NA objective. SARS-CoV-2 and influenza A motors are incubated with 10^{10} copies/mL of influenza A virus and only SARS-CoV-2 motors display long trajectories. The SARS-CoV-2 motors (6μm polystyrene) are distinguishable from the influenza A motors (5μm silica) on brightfield due to their darker outer rim. Scale bar is 10 μm.

Supplementary Movie S3: Timelapse video of the brightfield channel superimposed with color-coded particle trajectories acquired at 5 s intervals for a duration of 30 mins. The video was acquired following RNaseH addition using a 20x 0.5 NA objective. SARS-CoV-2 and influenza A motors are incubated with 10^{7} copies/mL of SARS-CoV-2 WA-1 and only influenza A motors display long trajectories. The SARS-CoV-2 motors (6μm polystyrene) are distinguishable from the influenza A motors (5μm silica) on brightfield due to their darker outer rim. Scale bar is 10 μm.

Supplementary Movie S4: Timelapse video of the brightfield channel superimposed with color-coded particle trajectories acquired at 5 s intervals for a duration of 30 mins. The video was acquired following RNaseH addition using a 20x 0.5 NA objective. SARS-CoV-2 and influenza A motors are incubated with 10^{10} copies/mL of influenza A virus and 10^{7} copies/mL of SARS-CoV-2 WA-1 and both motors remain stalled on the surface. The SARS-CoV-2 motors (6μm polystyrene) are distinguishable from the influenza A motors (5μm silica) on brightfield due to their darker outer rim. Scale bar is 10 μm.

Supplementary Movie S5: Representative timelapse smartphone video acquired at 5 s intervals for a duration of 15 mins using Cellscope. The video was acquired following RNaseH addition of DNA-based motors diluted in artificial saliva without virus present. Scale bar is 10 μm.

Supplementary Movie S6: Representative timelapse smartphone video acquired at 5 s intervals for a duration of 15 mins using Cellscope. The video was acquired following RNaseH addition of DNA-based motors incubated with 10^{7} copies/mL of SARS-CoV-2 B.1.617.2 spiked in artificial saliva. The motors were incubated with virus for 30 mins at room temperature and added to the Rolosense chip. Scale bar is 10 μm.

References

1. Kacherovsky, N. *et al.* Discovery and Characterization of Spike N-Terminal Domain-Binding Aptamers for Rapid SARS-CoV-2 Detection. *Angew. Chem.* **133**, 21381–21385 (2021).
2. Schmitz, A. *et al.* A SARS-CoV-2 Spike Binding DNA Aptamer that Inhibits Pseudovirus Infection by an RBD-Independent Mechanism**. *Angew. Chem. Int. Ed.* **60**, 10279–10285 (2021).
3. Zhang, Z. *et al.* A Universal DNA Aptamer that Recognizes Spike Proteins of Diverse SARS-CoV-2 Variants of Concern. *Chem. – Eur. J.* **28**, (2022).
4. Lai, H.-C., Wang, C.-H., Liou, T.-M. & Lee, G.-B. Influenza A virus-specific aptamers screened by using an integrated microfluidic system. *Lab Chip* **14**, 2002–2013 (2014).
